# FLASH radiotherapy: physico-chemical considerations on ionisation tracks, radical reactions, and the role of oxygen

**DOI:** 10.1186/s13014-026-02907-9

**Published:** 2026-08-20

**Authors:** Carsten Herskind, Peter O’Neill, Michael R. Horsman

**Affiliations:** 1 Cellular and Molecular Radiation Oncology Lab, Department of Radiation Oncology, Universitätsmedizin Mannheim, Medical Faculty Mannheim, Heidelberg University, Theodor-Kutzer-Ufer 1-3, 68167 Mannheim, Germany; 2 DKFZ-Hector Cancer Institute at University Medicine Mannheim, Heidelberg, Germany; 3 Medical Sciences Division, Department of Oncology, University of Oxford, Oxford, UK; 4 Experimental Clinical Oncology, Department of Oncology, Department of Clinical Medicine, Aarhus University Hospital, Aarhus, Denmark

**Keywords:** Ultra-high dose rate (UHDR), FLASH radiotherapy, Ionisation tracks, Radiation chemistry, Radicals, aqueous electrons, Time factors, Recombination, DNA, Chemical restitution, Oxygen fixation hypothesis, Mitochondria

## Abstract

The sparing effect of FLASH irradiation with ultrahigh dose rates (UHDR), by applying high dose fractions in less than one second, on adverse reaction in many normal tissues but not on tumour control has the potential to widen the therapeutic window and advance cancer radiotherapy. However, the mechanism is poorly understood, preventing its optimal application in the clinic. Here we review the current knowledge of radiation-induced radicals and their reactions, including time factors which are rarely considered. Furthermore, experimental in vitro and in vivo data in the framework of prevailing hypotheses, and the role of low oxygen concentrations and reoxygenation, are discussed. Finally, potential protective mechanisms are considered based on such reactions and the role of oxygen. We argue that more than one mechanism may contribute to dose-rate dependent protection, and that differences exist between protective mechanisms in vitro and in vivo. Furthermore, we propose a new protective reaction linking radiation chemistry to the role of oxygen in FLASH irradiation and incorporating the differential responses of normal tissue and tumours.

## Introduction

Irradiation with ionising radiation (IR) at ultra-high dose rates (UHDR: > 40 Gy/s) has received considerable attention since the rediscovery in 2014 of reduced biological effects compared with irradiation at conventional dose rate (CONV: typically <∼ 0.1 Gy/s) and naming of the sparing effect in normal tissue, but not tumours, the ’FLASH’ effect [1]. The prospect of decreasing normal-tissue reactions without compromising tumour control when compared to CONV radiotherapy (RT) promises a widening of the therapeutic window for tumour treatment that might take RT to a new level. The relative protection of normal tissues has been recapitulated in various preclinical models (for recent overviews, see [2–5]) although rarely in the same experimental models as the tumours. More recently, however, differential sparing effects within the same animal model were validated for early and late damage in tumour-bearing mice irradiated with FLASH protons or carbon ions [6–8]. In spite of these promising results and evidence from experimental studies in vitro and in vivo, the mechanistic basis of the reduced efficacy and the differential FLASH effect is still poorly understood. In particular, the relationship between UHDR effects in vitro and in vivo is not clear. Several publications have discussed the prevailing hypotheses which include radiation chemical and biological mechanisms, immune reactions, mitochondrial homeostasis, and long-lived proteins [3, 5, 9–18].

In general, dose-rate effects require interaction between two different ionisation events (tracks) separated in time to produce an effect that depends on the length of the time interval between them, the spatial distance and dimension of the tracks. Although complex radical reactions have been proposed to explain the FLASH effect [11, 12, 19], many reviews and original studies refer to basic radiobiological mechanisms in rather broad terms, for example, by referring to the role of reactive oxygen species (ROS) rather than specific, radiation-induced free radical species and their reactions. The purpose of the present review is to take a critical look at the early physico-chemical processes leading to relevant biological damage at ultra-high and conventional dose rates. Because most studies have been performed with sparsely ionising radiation (electrons and photons), and the bulk of the evidence points to a role of free radical reactions, we have limited our review to this radiation quality and focus on the two major hypotheses related to radiation chemical and biological mechanisms:

1. recombination of free radicals responsible for damaging DNA, leading to less damage to DNA (and other biomolecules);
2. transient oxygen depletion (TOD) leading to temporary local hypoxia with protection of normal tissue in contrast with tumours that already contain hypoxic areas.

## Radiobiology background

### Track structure, DNA damage and its repair

Ionising radiation is characterised by spatially correlated ionisations forming tracks in water, cells and other matter [20]. In aqueous solutions, high-energy (MeV) electrons produce tracks with typical linear energy transfer (LET) values around 0.2 keV/μm. Ionisations occur within picoseconds (ps) along the track consisting of single ionisations and clusters of 2–5 ion pairs (“spurs”) formed within a few nm. At electron energies >∼100 keV (LET <∼0.4 keV/µm), the spurs are well separated within the track (mean separation ∼500 nm) [21] but as fast electrons slow down to energies <10 keV, the distance between ionisations and spurs decreases and the residual kinetic energy of the electrons is deposited over a short range (10–30 nm for electron energies <∼1 keV) [22–24]. Because of the branching of electron tracks, about a third of the absorbed dose of MeV photon and electron beams is deposited by track ends of secondary electrons [25]. The importance of electron track ends for biological effects of low-LET radiation has been demonstrated by experiments with ultrasoft X-rays which deposit ∼100% of the dose in track ends and which show a relative biologic effectiveness (RBE) of approximately 3 but display all the features of low-LET radiations, including the radiosensitising effect of oxygen and the rejoining kinetics for DNA double-strand breaks (DSBs) [26, 27].

At the subcellular level, single ionisations may produce single base damage or DNA single-strand breaks (SSBs) while spurs, in addition to single lesions, may also result in DNA double-strand breaks (DSB), complex DSBs, and clustered DNA damage. The latter are also called Multiply Damaged Sites (MDS) and are defined by two or more non-DSB lesions (e.g., a SSB accompanied by a base damage) in the immediate vicinity of each other whereas complex DSBs are DSBs associated with further damage [28]. However, the major fraction of simple DSBs and complex DSBs with an additional SSB or base damage result from energy deposition events >100 eV whereas more complex DSBs with two closely spaced DSBs (and perhaps additional lesions) typically require >150–200 eV as deposited within a few nanometers in electron track ends [29]. The biophysical properties of track ends are characteristic of low-LET and are responsible for most of the complex damage causing the lasting biological effects of low-LET radiation. By contrast, the high-energy part of the electron spectrum predominantly produces non-clustered base damage and SSBs which are efficiently repaired by the base excision repair (BER) system including SSB repair.

It is important to emphasise that DSBs, complex DSB and clustered DNA damage sites are produced by single tracks. Two SSBs on opposite strands will result in a DSB only if they are formed within 10–15 bp from each other (corresponding to approximately a single 3.4 nm turn of the DNA double helix). If the distance between SSBs on opposite strands is larger, the probability that a DSB is formed decreases rapidly to zero, depending on G/C content and temperature. Microdosimetric considerations and Monte-Carlo (MC) simulations show that the probability of DSB induction by a combination of SSBs from two different tracks is negligible at doses <10^3^ Gy [22, 30]. This is supported by the SSB:DSB ratio which is only about 25 for cells irradiated with low-LET IR but 10^4^ for treatment with hydrogen peroxide (H_2_O_2_) at concentrations high enough to produce DSBs from randomly formed SSBs [31]. Consistent with this, experimental and theoretical approaches show that random SSBs produced by different ionisation tracks in cells, or by H_2_O_2_, are only close enough to form DSBs at radiation doses of several thousand Gy, or H_2_O_2_ concentrations producing SSBs equivalent to such doses [32, 33].

DSBs induced in an intact, double-stranded DNA helix are double-ended but if a break is induced at a stalled replication fork in S-phase, only one of the ends will be double-stranded while the complementary end will be single-stranded, thus constituting a single-ended DSB [34]. DSBs produced in single events as described above are considered lethal if left unrepaired. Low-LET radiation produces approximately 40 DSBs per Gy in a mammalian cell nucleus [35], but the mean number of lethal lesions per cell, m (calculated from the surviving fraction, SF = exp(-m), according to the Poisson distribution) is typically only 0.3–0.5 per Gy (corresponding to SF values between 0.74 and 0.59) so more than 98% of DSBs are rejoined for low-LET radiation. Repair after IR is part of the DNA Damage Response (DDR) which also comprises cell-cycle checkpoint activation and cell death/survival decisions, and in which the Ataxia Telangiectasia Mutated (ATM) protein kinase plays a central role. The two major DSB repair pathways are non-homologous end joining (NHEJ) which joins the ends rapidly but may introduce small deletions or insertions, and homologous recombination repair (HRR) which uses a sister chromatid as template and is error free. However, backup pathways such as alternative end joining and single-strand annealing exist which are more error prone [36].

Radiation-induced DNA repair foci are very sensitive markers of DSBs induced by moderate doses of radiation. Phosphorylated histone γH2AX marks regions of up to 2 Mb around a large fraction (50–60%) of DSBs and possibly other types of complex damage (e.g., MDS). The number of foci reaches a maximum at 30–60 min whereas a large fraction of DSBs are rejoined with faster kinetics (initial half-life ∼20 min) [37]. Since the choice and kinetics of the DSB repair pathway depends on the complexity of the DSB [38], simple DSBs that are rapidly rejoined may not be detected as γH2AX foci. Another important marker is TP53-binding protein 1 (53BP1) which marks DSBs but is essential only for the fraction of DSBs that are repaired with slow kinetics. This includes a subtype of NHEJ that requires limited resection by Artemis, and HR repair which requires more extended resection by EXO1 exonuclease [39]. Human 53BP1 plays an important role in regulating DSB repair pathway choice and is thought to be involved in relaxation of compact heterochromatin. It limits resection by Artemis and suppresses HRR in competition with BRCA which stimulates HRR [39, 40].

Unrepaired DSBs may represent more complex types of DSBs with adjacent base damage or SSBs, requiring more extensive processing. SSBs and base damage are also produced on a daily basis in normal cell physiology and are repaired very efficiently by the BER system. Notable exceptions are SSBs produced in S-phase during replication which may result in collapsed replication forks and give rise to single-ended DSBs during the repair process [41]. Thus, a small proportion of SSBs in proliferating cells may contribute to cell death if they result in unrepaired single-ended DSBs.

### Reactions of primary aqueous radicals: ^•^OH, e_aq_^−^, and ^•^H-atoms

Damage in DNA and other cellular biomolecules can result from direct action in which an ionisation event produces a radical site in DNA or the biomolecule, or from indirect action via attack by aqueous free radicals. Irradiation of water or dilute, aqueous solutions produces aqueous free radical species, hydroxyl radicals (^•^OH), hydrogen atoms (^•^H), and free electrons (e^−^) along the track, which subsequently react with each other in the spurs [42–44].

$$\rm IR \rightsquigarrow{H_2}O \to {H_2}{O^{ \bullet + }} + {\text{ }}{e^ - }$$

$$\rm IR \rightsquigarrow{H_2}O \to {H_2}{O^*}{ \to ^ \bullet }OH{\text{ }}{ + ^ \bullet }H$$

$$\rm {H_2}{O^{ \bullet + }} + {\text{ }}{H_2}O \to {H_3}{O^ + }{ + ^ \bullet }OH$$

$$\rm {e^ - } + {\text{ }}n{H_2}O \to {e_{aq}}^ - $$

$$\rm {e_{aq}}^ - + {\text{ }}{H_3}{O^ + }{ \to ^ \bullet }H{\text{ }} + {\text{ }}{H_2}O$$

$$\rm ^ \bullet OH{\text{ }} + {\text{ }}{e_{aq}}^ - \to O{H^ - }$$

$$\rm ^ \bullet OH{\text{ }}{ + ^ \bullet }OH \to {H_2}{O_2}$$

$$\rm ^ \bullet H{\text{ }}{ + ^ \bullet }H \to {H_2}$$

$$\rm {e_{aq}}^ - { + ^ \bullet }H{\text{ }} + {\text{ }}{H_3}{O^ + } \to {H_2} + {\text{ }}{H_2}O$$

$$\rm {e_{aq}}^ - + {e_{aq}}^ - + {\text{ }}2\ {H_2}O \to {H_2} + {\text{ }}2\ O{H^ - }$$

The initial yields in water (G-values, number per 100 keV or G ⋅ 0.10364 µM/J) at 1 picosecond (ps) are ∼5.0 for the ^•^OH radical and 4.1–4.6 for e_aq_^−^ [45]. During expansion of the ionisation tracks a proportion of the free radicals will react at early timepoints up to 100 nanoseconds (ns) under non-homogeneous kinetics^1^ through random collisions with each other or with biomolecules and solutes present in the immediate vicinity of the expanding track. At low scavenging capacity, radicals recombine inside spurs during track expansion to form hydrogen peroxide (H_2_O_2_), molecular hydrogen (H_2_), water (H_2_O), and hydroxide anions (OH^−^) [21, 46]. Track expansion is completed after approximately 0.1-1 µs when ^•^OH and aqueous (solvated/hydrated) electrons, e_aq_^−^ are homogeneously distributed [45, 47]. The stable yields are approximately 2.8 per 100 eV while the yield of ^•^H is much lower at 0.6 per 100 eV [44, 48] and the radicals which have survived track expansion now react under homogeneous kinetics. In the presence of oxygen, ^•^H and e_aq_^−^ react to form hydroperoxyl radicals (HO_2_^•^) and superoxide radical anions (^•^O_2_^−^) although it has been suggested that very little ^•^O_2_^−^ is formed in cells because many reactions compete with O_2_ for electrons under non-homogeneous reaction kinetics [12]. The ratio of indirect to direct action depends on the concentration of radical scavengers (scavenging capacity) present in the solution. For pure DNA in water, indirect action dominates and direct action can be neglected but with increasing scavenger concentrations, direct action becomes more important [44]. Because ^•^OH and e_aq_^−^ react with each other through a number of reactions, adding a scavenger of one species will typically reduce the decay rate, and thus increase the time-dependent yield, of the other species [21, 46, 49].

Inside nuclei of mammalian cells, DNA is organised as chromatin associated with histones and high concentrations of other proteins and biomolecules with high scavenging potential. Therefore, only aqueous radicals formed by radiation tracks very close to the DNA helix (e.g., within a distance of ~2 nm [35, 44]) are able to attack DNA (most of which will be scavengable damage) with non-homogeneous kinetics on a ns-time range along with direct ionisation of DNA (which cannot be scavenged by free radical scavengers).

The ratio of scavengable:non-scavengable damage is frequently used to estimate the ratio of indirect: direct action for the particular type of damage. For low-LET radiation under aerobic conditions, the ratio for immediate DSBs (before repair) was found experimentally to be 50:50 for ^60^Co γ rays [50] while, for clonogenic cell inactivation (radiobiological cell ‘death’), the ratio is frequently given as 2:1 or 3:1 [35, 51, 52]. For densely ionising, high-LET radiation, direct action dominates for cell inactivation, (i.e., the scavengable fraction of damage is small). The ^•^OH radical is by far the most reactive oxidative species. A number of studies estimated that it contributes 57% (range 44–60%) to lethal lesions under aerobic conditions [44, 48] while a more recent study found values of 76–85% [53]. The non-scavengable fraction will be due to unscavenged ^•^OH, other radical species, or direct radiation action. The ^•^OH radical reacts with almost any biomolecule in its immediate vicinity at essentially diffusion-limited rate under non-homogeneous kinetics which may affect experimental results as it is difficult to remove ^•^OH completely by adding external scavengers [54]. Using high scavenger concentrations, a lifetime of approximately 4–9 ns was estimated in mammalian cells, and a diffusion distance of 6 and 9 nm for SSBs and for cell killing, respectively, but the authors stated that these values may be overestimated [52]. An earlier study based on comparing the radiation sensitivity of wet and dry bacteria estimated a diffusion distance of 2 nm for inactivation [55] which would correspond to a lifetime of the order of 1 ns.

**Fig. 1:**
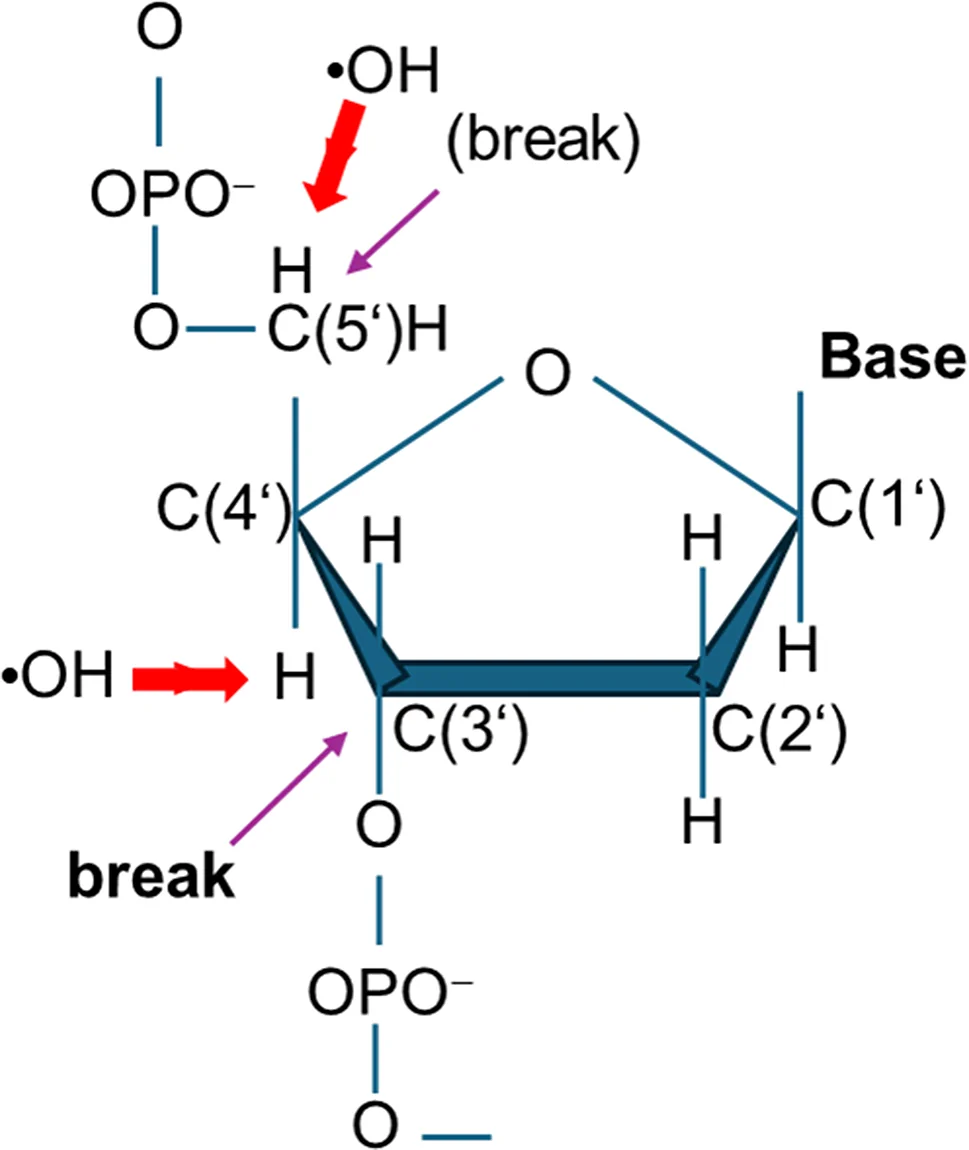
Major sites of H-abstraction by ^•^OH from C(5‘) and C(4‘) in deoxyribose radicals leading to a strand break at C(3‘) and more rarely C(5‘)

In cells, strand breaks are initiated either by direct ionisation of the DNA backbone or indirectly by an ^•^OH radical formed in close vicinity (within a few nm) of DNA. The ^•^OH radical abstracts an H-atom from deoxyribose, usually from C(5’) or C(4’) which are the outer, most accessible positions in the double-helix of double-stranded (ds) DNA [56, 57]. Subsequent intramolecular reactions of the deoxyribose radical then lead to breakage of the C(3’) or, more rarely, the C(5’) sugar-phosphate bond [44] (Fig. 1). In cells, potentially lethal DNA radicals that can be restituted by H-atom transfer (e.g., from thiols) before being fixed by oxygen have a decay half-life of the order of 1 ms which is five to six orders of magnitude longer than that of the DNA-damaging ^•^OH radical itself [58].

In addition to H-atom abstraction, ^•^OH radicals can also react with biomolecules by electrophilic addition to a carbon atom in C=C and C=N double bonds but not C=O in which the p-orbital electrons are more associated with the oxygen atom [44]. In single-stranded pyrimidine polymers (model compunds for RNA), a base radical formed by addition of ^•^OH to the C(5')=C(6') double bond in pyrimidine bases may lead to formation of an SSB by secondary H-abstraction from the adjacent sugar moiety [59]. The results indicated that the pyrimidine peroxyl radical formed by irradiation in the presence of oxygen may be more reactive than the base radical itself. In dsDNA, the bases are partly shielded by the double-helical structure but may be accessed from the major and minor grooves in B-DNA (the physiological conformation of DNA). Since the yield of base damage is higher than the yield of SSBs (approximately 2000–3000 versus 1000 per Gy) the possibility that ^•^OH attack on bases contributes to strand breakage in dsDNA cannot be discounted. Other reactions with bases lead to base damage and formation of abasic (loss of a base) and alkali-labile sites in dsDNA. DSB induction requires at least two ^•^OH radicals formed in the immediate vicinity of the 2.3 nm diameter DNA helix, or a combination of direct and indirect action, to cause breakage of both strands by the same ionisation event.

The ^•^H atom and e_aq_^−^ make up a weak acid-base system with pK_a_ = 9.1. Thus, at physiological pH (typically 7.2–7.6), less than 1% of ^•^H formed in ionisation tracks will be converted to e_aq_^−^. On the other hand, because of the low concentration of hydronium ions (H_3_O^+^, hydrated protons) the conversion of e_aq_^−^ to ^•^H (e_aq_^−^ + H_3_O^+^ → ^•^H + H_2_O) will be relatively slow [44, 60]. In contrast with its properties as a weak base, e_aq_^−^ is a strongly reductive species while ^•^H is slightly less reductive. In spurs, e_aq_^−^ may recombine with the ^•^OH radical to form OH^−^ but under high ^•^OH scavenger concentrations as in cells, the yield of e_aq_^−^ will be increased as described above. After track expansion, the lifetime of e_aq_^−^ in water depends on the concentrations of O_2_ and CO_2_ which capture e_aq_^−^ at nearly diffusion-limited rates. In aerated water, a lifetime of ∼160 ns is predicted [61] but this may extend to 300 µs in oxygen-free water [62, 63]. At high local concentrations (≥10^− 7^ M in water), e_aq_^−^ can recombine with itself to form molecular hydrogen: e_aq_^−^ + e_aq_^−^ + 2 H_2_O → H_2_ + 2 OH^−^ [63]. A dose of 1 Gy produces a cumulative concentration of 2.7⋅10^− 7^ M e_aq_^−^ so if this dose is given in a pulse length comparable to or shorter than the lifetime of e_aq_^−^, recombination between e_aq_^−^ from different tracks will be possible within and shortly after the pulse, before e_aq_^−^ has decayed. In cells, the high concentration of biomolecules acting as radical scavengers implies that the lifetime will be much shorter. However, reaction rates vary greatly depending on the type of molecular bond and the one-electron reduction potential [64], and thus concentrations and lifetimes of radiation-induced e_aq_^−^ might vary locally between different compartments (e.g., nuclei, cytosol).

The major reducing reactions of e_aq_^−^ with biomolecules are addition to a carbonyl group to form an anion radical species: R(C=O) + e_aq_^−^ → R^•^CO^−^, or through reactions involving transition metals or antioxidants [44, 64]. Thus, e_aq_^−^ reacts mainly with amino acids and polypeptides with reaction rate constants, k, increasing with the number of peptide groups but also being influenced by charge and other chemical groups [65]. Typical ranges of k are ∼10^7^–10^8^ M^−1^s^− 1^ for most amino acids, ∼10^8^–10^9^ M^−1^s^− 1^ for short peptides and ∼1-5⋅10^10^ M^−1^s^− 1^ for proteins [43, 65, 66]. By contrast, e_aq_^−^ is rather unreactive towards deoxyribose with a lower reaction constant of k = 1⋅10^7^ M^−1^s^− 1^ [43]. While it may react with nucleobases, in particular with pyrimidines which have a higher electron affinity, the bases will be shielded by the double-helical structure of DNA, and although addition to a phosphate group may cause a strand break in model compounds, e_aq_^−^ does not, in practice, contribute to DNA strand breakage [44]. If base damage is induced in DNA close to a DSB or SSB it contributes to more complex, or clustered damage which is more difficult to repair than simple base damage, SSBs and DSBs [67]. In addition to electron transfer in reduction reactions, ^•^H atoms can undergo electrophilic addition to C=C double bonds and abstract covalently bound H atoms from organic molecules (including DNA) leading to formation of molecular hydrogen (H_2_). However, the latter reaction is much slower than H-abstraction by ^•^OH [44]. Table 1 shows the major reactions of the primary radical species with DNA, different classes of biomolecules and redox agents.

**Table 1:** Major reactions of radiation-induced primary radicals and the superoxide anion radical with DNA, different classes of biomolecules and redox agents (e.g., transition metal cations or antioxidants such as glutathione, GSH)

Radical	DNA	Carbohydrates	Amino acids, proteins	Lipids	Redox/organic radicals
^●^OH	H-abstraction addition to C=C, C=N	H-abstraction	H-abstraction addition to C=C, C=N	H-abstraction addition.to C=C	oxidising (e.g. Fe_2_^+^, GS^−^)
^●^H	H-abstraction addition to C=O (bases)	H-abstraction	H-abstraction addition to C=C	addition to C=C	reducing (e.g. Fe_3_^+^, O_2_)
e_aq_^−^	addition to C=O (bases)	-	addition to C=O (e.g. histones)	-	reducing (e.g. Fe_3_^+^, O_2_); SET-PT
^●^O_2_^−^	-	-	-	addition to C=C (peroxidation)	oxidising towards radicals; removal by dismutation (SOD)

SET-PT: single-electron transfer followed by proton transfer. SOD: super oxidase dismutase

At early timepoints, reactions of electrons in the tracks under non-homogeneous kinetics will depend on random collisions with reactive groups in biomolecules and other solutes within the expanding track. The major protein components inside the nucleus are positively charged histones around which the negatively charged DNA helix is wound to form nucleosomes with a diameter of 11 nm. The string of nucleosome ‘beads’ is organised in a tertiary structure to form a 30 nm chromatin fibre although the existence and exact structure of the 30 nm fibre is debated [68]. After track expansion, reactions of the surviving electrons which are now hydrated e_aq_^−^ will be governed by diffusion and homogeneous kinetics. In cell nuclei, competition for e_aq_^−^ by reactive carbonyl (C=O) groups will be dampened because histones are shielded and electrically neutralised by DNA which itself shields the nucleobases in the double helix whereas the more exposed deoxyribose moieties do not compete for e_aq_^−^. Furthermore, although e_aq_^−^ react with free nucleotides at rate constants in the range of 10^8^-10^9^ M^−1^s^− 1^ [69], the nucleotide pool resides mainly in the cytoplasm with transporter proteins regulating the nuclear reservoir, keeping it at a low level except during replication [70]. Thus, the scavenging capacity for e_aq_^−^ in cell nuclei may vary with the cell cycle and be lower in quiescent cells.

The rate constant for the reaction of e_aq_^−^ with oxygen is k = 1.9⋅10^10^ M^−1^s^− 1^ [43] but the solubility of oxygen in water is relatively low so most electrons will react after track expansion, i.e., under homogeneous kinetics. Assuming an oxygen concentration, C, of approximately 230 µM in pure, aerated water (intermediate between the concentrations at 25 and 37 °C), the decay rate (k⋅C, equal to the scavenging capacity) at physiological conditions of 5% O_2_ will be λ = k⋅C = 1.05⋅10^6^ s^− 1^ and the corresponding lifetime τ = 1/λ = 0.96 µs. For moderately hypoxic conditions, 0.5% O_2_, the lifetime would increase correspondingly to 9.6 µs. Including the reactions of e_aq_^−^ with H^+^ and CO_2_ increases the scavenging capacity by ∼7% [61] and thus decreases the lifetime by a similar amount, In cells, ^•^OH radicals react rapidly in the tracks with non-homogeneous kinetics, thereby increasing the yield of e_aq_^−^, and oxygen may be consumed by reactions with radical sites in biomolecules (e.g., lipids), so the lifetime of e_aq_^−^ in the nucleus may well be long enough (≥1 µs) to survive track expansion, especially under partial hypoxia. Thus, e_aq_^−^ is likely to undergo reactions under homogeneous kinetics at some distance from the track where it was generated. Although calculations are complicated by the effect of macromolecular crowding [71, 72], such reactions may involve species generated by another track and thus potentially contribute to UHDR effects.

While most radiation chemical studies have been performed with model compounds or purified DNA in aqueous solution, studies on DNA inside cells are complicated by high concentrations of diverse biomolecules acting as endogenous radical scavengers. A model which is intermediate between the two is a single gene isolated in its transcriptionally active chromatin form. Multiple copies of the gene coding for ribosomal RNA (rRNA) are present in nucleoli isolated from the protozoan Tetrahymena where it exists as free, palindromic DNA with associated histones and RNA polymerases albeit in a more open structure than macromolecular (hetero)chromatin [73–76]. By irradiating this gene in dilute (1 mM) phosphate buffer, it was possible to study inactivation of transcription by free radical species under conditions where indirect action dominates although chromosomal proteins offer some local scavenging of radicals [77]. The inactivating lesions are mainly single-strand breaks and possibly abasic and alkaline-labile sites (but not base damage), causing blockage of chain elongation by RNA polymerases [77, 78]. Irradiation under N_2_, O_2_ or N_2_O (which converts e_aq_^−^ to ^•^OH) confirmed that ^•^OH and possibly the ^•^H atom but not e_aq_^−^ inactivate the biological activity and that no significant dose-modifying effect of oxygen was observed in the absence of added radical scavengers or sulphydryl compounds [77].

### Role of reactive oxygen species (ROS) and secondary radicals

Along with ^•^OH, H_2_O_2_ and ^•^O_2_^−^ are so-called reactive oxygen species (ROS) and are frequently claimed to cause cell death. H_2_O_2_ is produced by intra-track recombination of ^•^OH radicals in the spurs under heterogenous kinetics and, in dilute solutions at low scavenger concentration, it may also result from inter-track recombination under homogeneous kinetics after track expansion [42]. Alternatively, H_2_O_2_ is produced by dismutation of ^•^O_2_^−^ (see below). H_2_O_2_ is not very reactive by itself although it may react with radicals [44]. However, in the presence of transition metal ions, ^•^OH radicals may be formed in a Fenton-type reaction (e.g., Fe^2+^ + H_2_O_2_ → Fe^3+^ + ^•^OH + OH^−^). The ^•^OH radical is much more reactive than H_2_O_2_ and is responsible for most of its biological effects with DNA [44]. If ^•^O_2_^−^ is present, Fe^2+^ may be regenerated (Fe^3+^ + ^•^O_2_^−^ → Fe^2+^ + O_2_) thus enabling a chain reaction (ascorbate may also regenerate Fe^2+^ in vivo). However, because Fe^2+^/Fe^3+^, and long-lived H_2_O_2_ and ^•^O_2_^−^, are uniformly distributed by diffusion in aqueous solutions, the two reactions will not be spatially correlated. Therefore, the single ^•^OH radical formed from each H_2_O_2_ molecule will produce mainly random base damage and SSBs. At concentrations which produce similar amounts of SSBs as clinically relevant doses of ionizing radiation, essentially no DSBs or clustered damage will be formed since these require spatially correlated SSBs and/or base damage in close vicinity on opposite strands [31–33]. Consequently, DSBs only arise from random SSBs when their frequency is extremely high and the distance between neighbouring SSBs on opposite strands is less than 10–15 bp.

HO_2_^•^ and ^•^O_2_^−^ make up an acid-base system with pKa = 4.8 and thus approximately 0.3% will be present as HO_2_^•^ at physiological pH. ^•^O_2_^−^ is considerably less reactive than the ^•^OH radical with a life-time of milliseconds to seconds in aqueous solution [79, 80], implying that ^•^O_2_^−^ diffuses longer distances before reacting with other molecules or radical species. Thus, whereas ^•^OH reacts with other radicals in the tracks or with DNA under heterogenous kinetics, ^•^O_2_^−^ is formed under homogeneous kinetics after track expansion and diffuses longer, leading to a more uniform spatial distribution. At high pH, ^•^O_2_^−^ is quite stable but at low pH it reacts with HO_2_^•^ to form H_2_O_2_ and O_2_ in a spontaneous dismutation reaction which has a maximum rate when pH = pK_a_ [81]. At physiological pH, when ^•^O_2_^−^ is the dominant form, dismutation of two radical ions requires the enzyme superoxide dismutase (SOD). In mammalian cells, SOD exists in three forms associated with different transition metals: CuZnSOD (SOD1) located in the cytoplasm, MnSOD (SOD2) located in the mitochondrial matrix, and extracellular SOD (SOD3) which contains Cu and Zn and is secreted into the extracellular space. Thus, SOD1 is the important form for removing ^•^O_2_^−^ in nuclei. The SOD-mediated dismutation reaction is very efficient, occurring at a diffusion-limited rate, and thus depends mainly on the concentration of ^•^O_2_^−^ and the abundance of SOD. However, ^•^O_2_^−^ can also be reduced to hydrogen peroxide and other products by reaction with the ascorbate anion [44].

The oxidising properties of ^•^O_2_^−^ are mainly towards other radicals whereas it has low reactivity towards most biological molecules [82]. It undergoes addition reactions at C=C double bonds to form peroxy-radicals which, in lipids, can lead to chain reactions resulting in multiple peroxidation processes [44]. Although it is mainly an oxidising radical it also has reducing properties towards strong oxidants. Its toxic effects are considered to be related to lipid peroxidation and modification of biomolecules leading to ferroptosis and premature aging of cells [83]. Under normal physiological conditions, ^•^O_2_^−^ arises mainly by leaking from the oxidative phosphorylation process in the mitochondria where it is likely to cause toxicity by reaction with the membrane.

In spite of its oxidising properties, ^•^O_2_^−^ is practically unreactive towards DNA and its constituents [44]. Nevertheless, guanine base adducts have been described [84], and a study using KO_2_ as a source of ^•^O_2_^−^ found induction of SSBs (but not DSBs) in CHO hamster cells, which could be abolished by catalase and was reduced by a metal chelator [85]. This strongly implicated H_2_O_2_ in the reaction, suggesting that random SSBs were induced by ^•^OH radicals produced in a Fenton reaction from H_2_O_2_ formed by dismutation of the superoxide radicals. In contrast to the addition reactions of ^•^O_2_^−^, HO_2_^•^ is more prone to abstract an H atom from biomolecules but this reaction is slow and of minor importance at physiological pH where ^•^O_2_^−^ is the predominant form [44].

The Tetrahymena rRNA transcription system showed marginally less inactivation by irradiation under O_2_ than under N_2_, implying that ^•^O_2_^−^ plays no significant role in the induction of inactivating lesions [77] in agreement with the low reactivity towards DNA. Addition of ^•^OH radical scavengers (methanol, t-butanol, formate ions) under anoxia (N_2_) produced variable degree of protection which could be ascribed to inactivation by secondary scavenger radicals [86]. This is consistent with previous studies on inactivation of DNA by secondary radicals of phenylalanine, isopropanol and formate under anoxia [87, 88] and was later confirmed for DMSO-derived methyl radicals and secondary t-butanol radicals produced by reactions with ^•^OH [89, 90]. Under O_2_, all scavengers provided stronger protection against radiation-induced inactivation compared with under N_2_ and was independent of which scavenger was used [86]. The uniform protection under oxygen may be explained by inactivation of secondary scavenger radicals through formation of less reactive peroxidation products than secondary radicals under anoxia. These results further validated ^•^OH as the major inactivating species.

### The oxygen fixation hypothesis - modification of DNA radicals

The oxygen effect in vitro describes the observation that inactivation of mammalian cells to a given surviving fraction requires higher doses when cells are irradiated under hypoxic compared with oxic conditions. Thus, the oxygen enhancement ratio (OER) is defined as the ratio of doses required under hypoxic and oxic conditions to produce the same level of biological effect. Up to three times fewer lethal lesions per Gray are induced under hypoxia than under oxic conditions, corresponding to an OER of 3 for low-LET radiation. This is usually explained by the oxygen fixation hypothesis which states that radical sites in DNA produced by loss of an H-atom may be restituted under hypoxia by antioxidants such as thiol compounds present in cells, especially glutathione (GSH) which can donate an H atom from their sulphydryl (SH) group to the radical site in DNA in competition with fixation by oxygen (although steric changes may occur). If R represents DNA, then the two reactions compete:

$$\rm \begin{gathered} {{\mathrm{R}}^ \bullet }{\text{ + GSH}} \to {\text{RH + G}}{{\mathrm{S}}^ \bullet }{\mkern 1mu} \hfill \\ {\text{and }}{{\mathrm{R}}^ \bullet }{\text{ + }}{{\mathrm{O}}_{\mathrm{2}}} \to {\mathrm{R}}{{\mathrm{O}}_{\mathrm{2}}}^ \bullet \hfill \\ \end{gathered} $$

The rate constant of O_2_ with SSB and DSB precursor radicals is 2–3 orders of magnitude greater than that of GSH so oxygen outcompetes the antioxidants under aerobic conditions by forming a peroxy-radical which fixes the damage [35, 59, 91]. Using a gas-explosion technique, it was shown that mammalian cells must be exposed to oxygen within 5–10 ms after irradiation with an ultrashort 5 ns pulse of electrons in order for sensitisation to occur, consistent with a first half-life of the reactive DNA radical sites of approximately 1 ms in the absence of oxygen [58]. If oxygen is present during formation of the DNA radical, it may react faster with the DNA radical. As pointed out by Michael et al. [92], the fact that the radiosensitising effect of oxygen remains strong when O_2_ is added 0.1-1 ms after irradiation implies that molecular O_2_ is responsible for the oxygen effect and not the superoxide radical, O_2_^−^, formed in the reaction with e_aq_^−^ which has decayed by then (at >100 µs).

The radical site in DNA may be produced by H-atom abstraction but it cannot be excluded that loss of an electron in deoxyribose by direct action (RH→RH^+^ + e^−^) followed by dissociation of H^+^ (RH^+^ → R^•^ + H^+^) may produce a similar radical site. Furthermore, if the initial cation radical is in a base (e.g., guanine), an H atom may be abstracted from the adjacent sugar moiety producing a radical site that is amenable to fixation by oxygen or restitution by GSH [44, 59]. Of note, direct action by high-LET radiation (ions or neutrons) can interact with bound H-atoms producing energetic secondary H^+^ ions. The resulting DNA anion (R^−^) is not amenable to competition between fixation and restitution but may be neutralised by addition of a proton. However, the damage produced by the tracks of densely ionising radiation is usually too serious to be chemically restituted or repaired by the DNA repair system. By contrast, chemical restitution of parts of the lesions in MDS or complex DSBs produced by low-LET radiation might change a lesion from being lethal to being reparable by the DNA repair system thus rescuing the cell from lethality.

A recent study aimed to determine OER values for indirect and direct action by using high concentrations of DMSO and assumed that the non-scavengable fraction of damage represented direct action thus ignoring the effects of any residual ^•^OH radicals or secondary radicals. OER values of 2.01 and 1.32 were reported for direct action in CHO and AA8 hamster cells, respectively, and the overall OER for cell inactivation was interpreted as an average of these values and higher OER values for indirect action [53]. However, it is very difficult to scavenge ^•^OH completely since these react within a few ns under non-homogeneous kinetics. A further caveat of this and similar studies is that the potential role of secondary DMSO-derived radicals under hypoxia was ignored. In the case of DMSO, scavenging of ^•^OH produces a methyl radical which can react with itself leading to formation of C_2_H_6_, but also with DMSO and DNA [89, 93]:

$$\rm \begin{gathered} {\left( {{\mathrm{C}}{{\mathrm{H}}_{\mathrm{3}}}} \right)_{\mathrm{2}}}{\mathrm{SO}}{{\text{ + }}^ \bullet }{\mathrm{OH}} \to {\mathrm{C}}{{\mathrm{H}}_{\mathrm{3}}}{\mathrm{S}}\left({\mathrm{O}}\right){\mathrm{OH}}{{\text{ + }}^ \bullet }{\mathrm{C}}{{\mathrm{H}}_{\mathrm{3}}} \to {\left( {{\mathrm{C}}{{\mathrm{H}}_{\mathrm{3}}}} \right)_{\mathrm{2}}}{\mathrm{S}}{{\mathrm{O}}_{\mathrm{2}}}^ \bullet{\text{ + C}}{{\mathrm{H}}_{\mathrm{4}}} \hfill \\ \end{gathered} $$

$$\rm RH{ + ^ \bullet }C{H_3} \to {R^ \bullet } + C{H_4}$$

Inside cells, the sulphydryl antioxidant GSH may donate an H-atom to any of these radical sites under hypoxia making predictions for the competing reactions between scavenger radicals, oxygen and H-atom donation very complicated indeed. In the Tetrahymena chromatin system where indirect action dominates and secondary scavenger radicals were important under N_2_ [86], it was shown that OER values of about 2 were obtained in the presence of moderate concentrations (0.1-1 mM) of the thiol compound, mercaptoethanol. However, this OER value decreased to 1 or even less when 10 mM or 300 mM t-butanol was added [94]. Thus, OER values for indirect action can be modulated by different combinations of sulphydryl and scavenger concentrations. Therefore, interpretation of OER from results using DMSO or other ^•^OH radical scavengers to eliminate scavengable damage under hypoxia should be viewed with caution.

The oxygen fixation hypothesis is supported by most evidence with half maximum OER values usually observed at approximately 3 mmHg (0.4%, 4–5 µM O_2_) [35]. However, a biphasic OER curve has been described in a study on V79 hamster cells with half maximum effect at approximately 1 µM O_2_ for the first component and at approximately 30 µM for the second component and with a flat response in the range 1.5-7 µM [44, 95]. Furthermore, a reduced OER value of 1.5 for cell survival and 1.2 for SSBs was reported for GSH^−/−^ null mutant cells compared with 2.9 and 3.0, respectively, for GSH^+/+^ wildtype cells [96]. However, to the best of our knowledge, no further studies have reproduced these findings nor identified a mechanism for the biphasic behaviour.

In addition to thiols, other antioxidants and naturally occurring free radicals such as Vitamin C (ascorbic acid, AscH_2_) and Nitric Oxide (NO) may take part in the competition between fixation and molecular restitution of radicals induced in DNA and other biomolecules. These molecules are more likely to play a role in vivo as they are usually not present in cell culture medium in vitro.

Vitamin C is a water-soluble antioxidant that is important for the regeneration of other antioxidants (e.g., fat-soluble vitamin E) and functions as a cofactor of enzymes, such as in collagen synthesis. It is synthesised in the kidneys or liver of many animal species but not primates and certain rodents (e.g., guinea pigs) which rely on external sources [97]. Addition of vitamin C was able to reduce DNA damage in vitro induced by treatment of DNA in solution or in mammalian cells with IR or H_2_O_2_ [98–100]. Furthermore, physiological ascorbate levels or oral vitamin C supplements given to untreated human donors in vivo showed protection against oxidative DNA base damage and strand breaks in lymphocytes or peripheral blood mononuclear cells (PBMCs) treated ex vivo [101–103]. Mouse experiments showed radioprotection of normal intestine by pharmacological ascorbate given at 4 mg/g bodyweight while tumours were radiosensitised [104]. A recent study confirmed these findings in a different murine system and showed a correlation with TGF-β suppression for normal tissue protection [105]. In the same study, an increased H_2_O_2_ flux led to fixation of ATM as inactive homodimers in the cytoplasm of tumour cells causing radiosensitisation by interfering with the DDR program.

These different studies were performed in aerated conditions so the protective effect occurred even at ambient oxygen concentration. However, low oxygen concentrations would be expected to favour H-atom transfer from ascorbate (leaving an ascorbate radical anion, Asc^• −^) to a C-centered radical in DNA [106, 107] relative to fixation by oxygen (R^•^ + AscH^−^ → RH + Asc^• −^). Thus, physiological concentrations of ascorbate may conceivably be important for restitution under hypoxia but more studies will be needed to clarify its role under hypoxia and potential influence on the OER in vivo.

NO is a free radical (actually ^•^NO) which plays important roles as a signalling messenger in vasodilation and the function of various tissues including muscles, brain, and the immune system [108]. It is relatively stable under physiological conditions but may produce a cell-toxic oxidant, the peroxynitrite anion (ONOO^−^) by reaction with ^•^O_2_^−^ (^•^NO + ^•^O_2_^−^ → ONOO^−^) [109]. NO reacts extremely fast with other radicals, for example, in proteins or DNA (R^•^ + ^•^NO → RNO), thereby acting as an antioxidant [110, 111]. The NO adduct produced by addition to radiation-induced DNA radical sites makes NO a radiosensitiser by fixing the damage in a similar way as oxygen but reacting even faster [112, 113]. Furthermore, it was shown to react with deoxyguanosine monophosphate to produce two modified nucleoside monophosphate products [114]. When plasmid DNA was irradiated, this reaction protected the yield of prompt SSBs while increasing the yield of base damage that were sensitive to glycosylase and thus in principle repairable by BER. Further experiments with cells suggested that if this type of damage were present in clustered damage sites, it would make repair more difficult and result in DSB formation at stalled replication forks during replication in the S phase [114]. More work will be needed to establish the contribution of NO to DNA damage fixation in vivo in different tissues.

## UHDR studies in vitro and in vivo

### Early studies with single pulses: radical recombination or oxygen depletion?

UHDR irradiation was first studied in vitro in the 1960s by Town who found that 15 MeV electrons given as a single 1.3 µs pulse caused much less incremental inactivation of HeLa-S3 cells in the dose range 10–30 Gy than at doses up to 9 Gy [115]. The effect disappeared if the same doses were given in two pulses separated in time by 2.5 ms. Because the survival curve at higher doses was parallel with the more resistant survival curve under nitrogen which did not show a dose-rate effect, it was suggested that radiation-induced hypoxia might be responsible for the UHDR effect. On the other hand, a study by Todd et al. found no sparing effect up to 9–10 Gy in a human kidney cell line irradiated with a much shorter pulse (30 ns) of 10 MV X-rays [116]. Under aerated conditions, the cells even showed increased sensitivity to UHDR at 10–11 Gy compared with Co-60 γ-rays at conventional dose rate at doses up to 13 Gy. A normal oxygen effect was observed for pulsed X-rays with no upward breakpoint of the survival curve up to 10 Gy under air or 30 Gy under nitrogen (∼anoxia) but doses higher than 10 Gy were not tested under aerated conditions [116]. However, a subsequent study by Berry et al. [117] found a reduced slope of the survival curve of a hamster lung cell line and HeLa-S3 cells above 5 Gy for a 7 ns pulse of 2 MV X-rays. By contrast, the same study found no change for a 50 ns pulse of 3.7 MV X-rays whereas 19 ns caused a breakpoint in the slope at 10–12 Gy increasing to 25 Gy for a 35 ns pulse. These authors considered it unlikely that a dose of 5 Gy could cause local oxygen depletion and suggested recombination of radiation-induced radicals as a more likely cause for the sparing effect of UHDR. However, no calculations were performed to substantiate this mechanism and the results were not confirmed in a subsequent study by Berry and Stedeford [118].

### The role of radical-radical recombination

As described earlier, recombination of primary ^•^OH radicals produced within a single ionisation track cannot contribute to dose-rate effects since this requires interaction between events in different tracks produced with a time interval between them. A well-established example of a dose-rate effect at conventional dose rates is split-dose recovery in the time interval between two dose fractions or during continuous low-dose rate irradiation. It takes place on a time scale of several minutes and is ascribed to repair of a sub-lesion before a second sub-lesion is formed, thus preventing interaction between the two sub-lesions to form a lethal lesion.

However, effects of UHDR must involve much faster interactions in the ns-to-ms time range. In water or dilute solutions, ^•^OH radicals produced in different tracks at UHDR may in theory survive track expansion and recombine with radicals from different tracks formed within their lifetime and diffusion distance. Thus, H_2_O_2_ might be expected to increase for UHDR owing to increased inter-track recombination of ^•^OH radicals. However, levels of H_2_O_2_ were shown to decrease rather than increase after irradiation with UHDR compared with CONV, irrespective of whether pure water or an aqueous solution of 4–5% albumin (to provide a scavenging capacity equivalent to that of extracellular albumin in blood) were irradiated [119–121]. The decrease of H_2_O_2_ yields in experimental studies of UHDR versus CONV dose rates has been ascribed to reduced recombination of ^•^OH radicals caused by increased competition for ^•^OH by e_aq_^−^ [122]. However, inter-track recombination of these radicals may only be important in dilute solutions where the OH scavenging capacity is low and not in 4–5% albumin with an ^•^OH scavenging comparable to that in blood serum. Another contribution to H_2_O_2_ in dilute solution may be from dismutation of superoxide radicals (^•^O_2_^−^) produced by capture of e_aq_^−^ by oxygen in aerated solutions. Under these conditions, ^•^O_2_^−^ have an extended lifetime which may compensate for the rather slow spontaneous dismutation reaction in the absence of SOD [44]. Thus, another explanation for the lower production of H_2_O_2_ after UHDR in the experiments described above [119, 120, 122] might be a shift from electron capture by oxygen towards a competing inter-track recombination reaction such as e_aq_^−^ + e_aq_^−^ + 2 H_2_O → H_2_ + 2 OH^−^ and e_aq_^−^ + ^•^OH → OH^−^ in dilute aqueous solution [63] resulting in less ^•^O_2_^−^ being formed. The former reaction might explain why radiation-induced H_2_O_2_ is also lower at UHDR in the presence of albumin as an ^•^OH scavenger.

Importantly, there is a discrepancy in H_2_O_2_ yields for UHDR relative to CONV dose rates between the results obtained in recent experimental studies on water showing mostly a reduction, and Monte Carlo simulations showing mostly an increase [123]. However, non-homogeneous reactions of radicals during track expansion in the time interval 1 ps − 1 µs are difficult to model and require a number of assumptions, including the recombination of radicals between tracks (inter-track interaction) as reviewed by Shiraishi et al. [124].

Whereas overlap of expanding tracks may be an issue in pure water where free radical lifetimes are long enough to survive track expansion and allow diffusion of radical species between different tracks, ^•^OH radicals react very fast with heterogenous kinetics inside cells. The lifetime and diffusion distance are short (≤4–9 ns and ≤6–9 nm, respectively) [52] implying that recombination occurs only in spurs within individual tracks (intra-track interaction) before full expansion at ∼0.1 µs. The distance between nearest neighbour tracks may be estimated by a semi-quantitative calculation. A dose of 1 Gy produces approximately 1000 tracks in a nucleus with an average of 100 ionisations per track [35, 125]. Assuming a cross section of 50 µm^2^ for the nucleus, the average area per track is 0.05 µm^2^ which can be approximated by a circular area, A = π ⋅ r^2^ where r is the diameter. The average distance between neighbouring tracks will be about 2r which is approximately 250 nm for a dose of 1 Gy. A slightly more accurate estimate assuming hexagonal packaging is only marginally smaller at 240 nm. For doses of 0.25–5 Gy, the inter-track distance would be approximately 500 nm and 100 nm, respectively. In order to produce track separations of ∼8 nm or smaller that could overlap within the lifetime of the ^•^OH radical, a dose of 1000 Gy would have to be given in less than 10 ns, corresponding to a dose rate in the pulse of >∼ 10^11^ Gy/s. This conclusion is supported by Monte Carlo-modelling of laser-accelerated proton tracks, where a minimum dose of 23–26 Gy was required for overlap of different tracks of 80–100 MeV protons (LET = 0.7–0.9 keV/µm) at 1 ns [126, 127]. Such high dose rates are not achieved with standard electron beams currently used for FLASH irradiation and thus inter-track recombination of primary ^•^OH radicals to form H_2_O_2_ cannot explain the protective effect of UHDR/FLASH with electrons giving 1–5 Gy per 1–5 µs pulse. Much higher dose rates may be achieved with laser plasma accelerators giving 10–15 Gy in a single ∼30 femtosecond pulse [128].

Several studies with a single UHDR pulse were unable to reproduce the break in the slope of the survival curves under air first described by Town and by Berry et al. [115, 117]. This was the case for different human and hamster cells irrespective of the pulse length ranging from 3 ns to 5 µs: 3–10 ns with HeLa and CHO cells [118, 129–131], 30 ns with human kidney cells [116], 1–5 µs with HeLa cells [132], 3 µs with U87 human glioblastoma and HT-144 melanoma cells [133]. Furthermore, none of the studies showed a breakpoint in the slope of the survival curves under anoxia (N_2_) which would have been expected for pulses in the ns range if inter-track ^•^OH radical recombination was responsible for the induced protection. Even when ultrashort pulses (< 10 ps) of ultrasoft X-rays (mean energy 1.2 keV) were used at a repetition rate of 5 pulses per second (pps), doses in the range 2–12 Gy showed typical low-LET survival curves under air and N_2_ with an OER of 2.4, without evidence of a breakpoint (a breakpoint at higher doses, caused by partially shielded cells, was excluded) [26]. However, the repetition rate and the dose per pulse (DPP = 0.07 Gy) were rather low resulting in dose delivery times of 6–34 s which do not qualify this UHDR source as FLASH radiation.

Taken together, a decrease in the level of the inactivating radical species (^•^OH) owing to increased recombination of primary free radicals is unlikely to explain protection observed at UHDR. Furthermore, this mechanism would not explain the differential sparing effect of FLASH in normal tissue relative to tumours.

### The role of radiation-induced oxygen consumption

Radiation-induced oxygen depletion leading to transient hypoxia was proposed as a protective mechanism of UHDR irradiation for cell inactivation in vitro and different in vivo endpoints [115, 134–136]. Therefore, when differential protection of normal tissue relative to tumours by FLASH irradiation was first described, transient local oxygen depletion was suggested as an explanation for protection in normal tissue whereas it was assumed to have less effect in tumours which frequently contain hypoxic areas [1].

In aerated aqueous solution, oxygen is consumed by capturing e_aq_^−^ leading to formation of O_2_^−^. In irradiated cells, however, biomolecules (in particular polypeptides) compete for e_aq_^−^ [12, 44, 137].

$$\rm {O_2} + {\text{ }}{e_{aq}}^ - {{\text{ }} \to {\text{ }}^ \bullet }{O_2}^ - $$

$$\rm R\left( {\mathrm{C=O}} \right){\text{ }} + {\text{ }}{e_{aq}}^ - \to R({C^ \bullet}-{O^ - })$$

The reaction rate constants are k = 1.9⋅10^10^ M^−1^s^− 1^ for electron transfer to oxygen [43] and, as previously discussed, k ∼ 10^8^ − 5⋅10^10^ M^−1^s^− 1^ for the reaction with C=O in polypeptides [43, 65, 66]. During irradiation, C-centered radicals in biomolecules are formed by H-atom abstraction or by addition of ^•^OH radicals to C=C double bonds [44, 138].

$$\rm RH{{\text{ }} + {\text{ }}^ \bullet }OH \to {R^ \bullet } + {\text{ }}{H_2}O$$

$$\rm \left( {\mathrm{C=C}} \right){{\text{ }} + {\text{ }}^ \bullet }OH{\text{ }}{ \to {\text{ }}^ \bullet }C - C\left( {OH} \right)$$

The reaction rate constant for O_2_ with C-centered radicals is 2⋅10^9^ M^−1^s^− 1^ [44] but, in lipids (such as found in membranes), a chain reaction of peroxidation may increase the oxygen consumption [139].

$$\rm {R^ \bullet } + {\text{ }}{O_2} \to R{O_2}^ \bullet $$

$$\rm R{O_2}^ \bullet + {\text{ }}RH \to R{O_2}H{\text{ }} + {\text{ }}{R^ \bullet }$$

Because of the high intracellular concentration of biomolecules, it has been suggested that e_aq_^−^ is more likely to be scavenged by biomolecules than to react with oxygen [12]. Therefore, intracellular oxygen consumption may be due mainly to reactions with radiation-induced radicals on biomolecules leading to formation of peroxidation products rather than direct reaction of O_2_ with e_aq_^−^ leading to formation of superoxide radicals.

Early studies of radiation-induced oxygen consumption using oxygen probes (electrodes) yielded 430 ppm/Gy (0.49 µM/Gy at 27 °C) in cell culture medium under 0.5–1.1% oxygen [140] (corresponding to pO_2_ = 3.7–8.2 mmHg) and 0.44 µM/Gy over a wide range (0.1–20.9%) of initial oxygen concentrations [141]. A more recent experimental study using a dendritic phosphorescent probe found 0.28–0.30 µM/Gy for UHDR with no apparent influence of initial oxygen concentrations (partial pressure) of 8–9 and 21–22 mmHg, respectively [142]. For the same dose, the total consumption was slightly lower when given at UHDR than for CONV but because of the short dose delivery time the rate of consumption was much faster for UHDR. Since control experiments showed similar readings of the phosphorescent probe and an oxygen electrode, the lower G-values did not appear to be related to the measurement method. Another experimental study, and model simulations in water in the absence or presence of free-radical scavengers, also indicated a somewhat smaller oxygen consumption of 2–4 µM after a dose of 20 Gy [142–144], corresponding to 0.1–0.2 µM/Gy. However, recent studies on water containing 4% albumin found a linear dependence of O_2_ consumption on the initial oxygen concentration below 20 mmHg with approximately 0.25 mmHg/Gy at 10 mmHg and 0.45 mmHg/Gy at 20 mmHg, reaching a maximum at initial concentrations higher than 30–50 mmHg [120]. This corroborated the response after UHDR irradiation with protons [145] which also showed an approximately 30% decrease in the consumption when the dose rate was increased from 0.5 Gy/s to 100 Gy/s [146]. Notably, little or no difference between irradiation at UHDR and conventional dose rate was observed at these low initial concentrations but O_2_ consumption reached a lower constant level of 0.45 mmHg/Gy after UHDR compared with 0.65–0.7 mmHg/Gy after CONV. These studies were performed in aqueous solutions with albumin at relative low concentration as a radical scavenger, which is uniformly distributed and differs considerably from the densely packed biomolecules in cell nuclei. Nevertheless, a similar dependence of oxygen consumption on the initial oxygen concentration was found for proton FLASH in muscle interstitial space in vivo [145, 146], inside mammalian cells in vitro [147], and in mouse skin [148]. Thus, FLASH doses of 10–30 Gy may be able to partially deplete oxygen by 20–60% of the initial O_2_ concentrations in the range up to 10–20 mmHg while 4.5–13.5 mmHg may be depleted at higher concentrations. A caveat is the difference in dependence on initial oxygen concentration between the earlier and the most recent studies. Since the phosphorescent probes contain a porphyrin ring which may act as an electron donor or acceptor [149] it might be interesting to test if the molecular oxygen sensor may compete for radiation-induced e_aq_^−^ at low oxygen concentrations. On the other hand, measurements using a fibre-optic oxygen sensor incorporating a fluorescence probe (ruthenium (II) chloride complex) at the tip showed a similar dependence on low initial oxygen concentrations [150].

Interestingly, a recent study found that the chemical environment influences radiolytic oxygen consumption and that ascorbate decreased the rate and imposed a dependence on low concentrations [151]. Normally this should not be relevant for cell irradiation in vitro as ascorbate is missing from standard cell culture media but it may be relevant in vivo.

As a technical note, cell culture plasticware, such as polystyrene vessels frequently used to grow cells in vitro, may release small amounts of oxygen during the experiment. Thus, it has been clearly demonstrated that the O_2_ concentration in the immediate vicinity of attached cells may be increased sufficiently to radiosensitise the cells under N_2_ [58, 152].Therefore, cells should be irradiated in glassware or Permanox^®^ under hypoxic conditions [153]. However, dose corrections to account for increased backscatter must be applied when using glass.

### The role of low oxygen concentrations: studies on cells in vitro

In contrast with the studies above, an induced change in slope of survival curves with a breakpoint at approximately 7 Gy was reported when cells were irradiated under low oxygen tension (0.35% = 2.7 mmHg) with a 1 µs or a 3 ns pulse [118, 132]. This suggested a critical role of low oxygen concentrations for the sparing effect of UHDR which was confirmed by Epp et al. demonstrating changes in the final slope for oxygen concentrations in the range 0.26–0.91% O_2_ (2–7 mmHg) but not under air or N_2_ in HeLa cells irradiated with 3 ns pulses of 350 keV electrons [129]. A similar result was found for 0.44% O_2_ (3.4 mmHg) by Michaels et al. after irradiation with 3 ns pulses of 450 keV electrons [130]. Furthermore, the latter study showed that the parallel final slopes of the curves were shifted linearly as a function of the oxygen concentration in the range 0-0.7% (0-5.5 mmHg). In bacteria, similar break points and parallel final slopes had been observed at 100–300 Gy for oxygen concentrations ≤8% but not under air [154]. The higher doses were required because of the much smaller size of the E. coli bacterial genome. Together, these studies emphasise the importance of low oxygen concentrations for the protection of cells irradiated at UHDR.

The divergent results under air in the two early studies [115, 117] may have been caused by low oxygen concentrations induced by the experimental protocols prior to cell irradiation. It is known that the cell metabolism consumes oxygen and may cause hypoxia under certain experimental conditions [155]. For example, in preparation for a radiobiological RBE study [156], pilot experiments showed induced radiobiological hypoxia when high numbers of cells (10–20 ⋅ 10^3^) were kept in pellets for 1 h before irradiation with doses >10–15 Gy at conventional dose rate [Q. Liu and C. Herskind, unpublished data]. Similarly, a hypoxic subpopulation may give rise to a breakpoint in the survival curve [12]. In the experiments by Town [115], cells were irradiated in suspension in polystyrene bottles but further details regarding volumes and cell densities were not given. In the work by Berry et al., flasks with plated cells were equilibrated with air/5% CO_2_, sealed, incubated overnight, then transported to the irradiation site where they were irradiated 20–24 h after plating [117]. In both cases, high cell numbers would have been used to detect colony formation by surviving cells after higher doses. This might have caused hypoxia, although in the second series of experiments by Town [115], air or N_2_ was bubbled through the suspension. However, cells may have sedimented in the absence of agitation.

It should be noted that in the studies where no breakpoint was observed, monolayer cells were irradiated either in vertical dishes without cell culture medium (single 3 or 30 ns pulse lengths) [116, 129, 130] or horizontally with medium (10 ns, 1 µs or 3 µs pulse lengths) [131–133]. Thus, there was no evidence for hypoxic fractions of cells in either of these configurations.

A previous study showed no sparing effect of UHDR relative to CONV on survival of V79-379 hamster lung fibroblasts at doses up to 15 Gy under air or up to 39 Gy under hypoxia [157]. Using more sensitive human pancreatic cell lines, a more recent study even found an enhanced sensitivity up to 8 Gy after UHDR irradiation under air at a mean dose rate (MDR) of 35 Gy/s which is at the lower limit of FLASH dose rates [158]. However, in another study, different human tumour cell lines showed protection by UHDR at doses up to 9 Gy under air whereas no protection relative to conventional dose rate was observed for MRC5 fibroblasts [159]. In the relative radioresistant DU145 human prostate cancer cell line, a sparing effect of UHDR compared with CONV was found in the dose range 12–24 Gy at low oxygen concentrations in the range 1.6–4.4% [160]. No breakpoint in the slope of the survival curve was seen but it was argued that complete depletion of oxygen would require even higher doses.

Model simulations of spheroids showed an increased diameter of the hypoxic core by irradiation at FLASH but not conventional dose rate when constant replenishment of oxygen was assumed [161]. The hypoxic core was visualised experimentally and the presence of a radio-biologically hypoxic subpopulation was supported by increased radio-resistance of A549 cancer cells with breakpoints in the survival curves at approximately 6 Gy for FLASH given at a rate of 90 Gy/s and 8 Gy for conventional dose rate. This was consistent with an increase in the hypoxic fraction of cells by UHDR irradiation and resulted in a dose modifying factor for FLASH which increased with dose to a maximum of 1.3 at approximately 16 Gy.

### The role of low oxygen concentrations: in vivo studies

Early studies on normal tissue in vivo showed a sparing effect of UHDR in mouse intestines after whole-body irradiation with 7 MeV electrons (2 µs pulses at 400 pps) at 16.7–83 Gy/s versus 1 Gy/s [136]. When the animals were breathing oxygen, UDHR doses of 13.4 and 16 Gy (0.17–0.96 s irradiation time) resulted in 50 and 90% death, respectively, whereas lower doses (11.9 and 14 Gy) produced the same effect when applied at the lower dose rate (12–14 s duration). The dose-rate effect was dependent on oxygen and disappeared when the animals were breathing nitrogen (the lower dose rate was increased to 2.5 Gy/s to keep the time of nitrogen breathing short). This was ascribed to a radiation-induced decrease in oxygen tension but the high OER of ∼2.8 observed at low dose rate suggests that full sensitisation by oxygen was achieved, indicating that the intestinal tissue under oxygen breathing was well oxygenated and not already partially hypoxic. A subsequent study on local irradiation of rat hind feet showed clear protection for early and late skin reactions and for deformity after irradiation at 67–83 Gy/s (strong protection) compared with 2 Gy/min (0.033 Gy/s) or 1 Gy/s (non-significant protection) under air breathing [135]. Irradiation under anoxic conditions required higher doses but showed no sparing effect of the high dose rate. It was concluded that the sparing effect could be explained by radiation-induced consumption of oxygen if the initial concentration was about 7 mmHg (approximately 0.9%) in skin. However, this estimate was based on the observed sparing of early skin reaction at doses >20 Gy and an O_2_ consumption of 0.2 mmHg/Gy, but no direct measurements were performed to support this interpretation.

In a seminal study by Hendry et al. [134], mouse tail necrosis after irradiation with 10 MeV electrons given in 0.5-5 µs at a rate of 1–50 pps was tested for wide ranges of doses per pulse and dose rates within the pulse. Maximum protection by UDHR irradiation was observed for doses ≥0.2 Gy per pulse and dose rates >10^5^ Gy/s within the pulse when the animals were breathing air. Irradiation of clamped tails was slightly more protective than for UHDR under air but was the same for low and ultrahigh dose rates. Further experiments showed increased sensitivity of the tails when irradiated in 37 °C warm air compared with room temperature (21–25 °C) or when the mice were breathing oxygen [134]. By contrast, irradiation at 19–23 °C was nearly as protective as clamping the tails, strongly suggesting that the mouse tails were neither fully oxygenated nor completely anoxic under normal physiological conditions and that perfusion was reduced at the lower temperature. Based on a constant rate of radiation-induced oxygen depletion, initial oxygen concentrations in the range 3.3–6.1 µM (corresponding to approximately 0.3–0.6%) were estimated. Modelling reoxygenation is not straightforward but a first estimate of diffusion times as a function of distance, x, may be obtained from the Einstein-Smoluchowski equation for diffusion in three dimensions,

$$\rm \tau = {x^2}/(6 \times D)$$

where D is the diffusion constant. In water, the diffusion constant for oxygen is approximately 2⋅10^− 5^ cm^2^/s but it may be 2-6-fold smaller in tissues [162]. Therefore, diffusion times for a distance of 50–100 μm may be expected to be in the range 0.4–5 s. The critical upper limit of 0.5–1 s for the dose delivery time to achieve FLASH protection is consistent with the lower value of this range while the time of approximately 5 s for experimental reoxygenation of interstitial fluid in mouse thigh muscle after FLASH irradiation [146] is consistent with the upper value. The protective UHDR effect on rat tail necrosis was observed for irradiation times up to 4.5 s, so it seems possible that reoxygenation may be determined by diffusion although it cannot be excluded that changes in perfusion of the vessels contributes to regulation. Intriguingly, some increase in protection, as a function of dose rate in the pulse, was observed in the intermediate range from 3⋅10^3^ Gy/s to 6⋅10^4^ Gy/s where the total irradiation time was ≥20 s and up to several minutes. This may indicate a component of the protective UHDR effect which depends on the dose rate within the pulse (at least for pulse lengths in the range 0.5-5 µs) but is independent of the total irradiation time.

More recent evidence that physoxia (∼1–8% O_2_) is essential for the relative protection of normal tissue by UHDR with 6 MeV electrons comes from irradiation of mouse brains. Thus, neurocognitive sparing was markedly reversed when the oxygen concentration was increased by breathing carbogen (95% O_2_ + 5% CO_2_) instead of air [119]. Furthermore, a protective FLASH effect was demonstrated for acute skin toxicity in a mouse foot model under physioxia (17–28 mmHg; ∼2.2–3.7%) but not under hypoxia (2-7.5 mmHg; ∼0.2-1%) induced by clamping the leg [163]. Overall, the results from in vitro and in vivo studies under controlled oxygen conditions reviewed above strongly support a dependence of UHDR/FLASH sparing effects on oxygen, with most of the evidence pointing towards a requirement for low oxygen concentrations, 0.26–4.4% for cells in vitro, and ∼0.3–0.5 to 8% O_2_ for tissue in vivo. In this context, it should be noted that some evidence suggests that the oxygen concentration in mitochondria and cell nuclei may be lower than in the cytosol [164, 165] and that this may be explained by the so-called ‘Hoover^®^’ effect [166].

### Protective effects of UHDR on DNA damage induced by irradiation of DNA in solution

Induction of DNA strand breaks by FLASH irradiation has been studied on plasmid DNA in aqueous solution. The plasmid suspension was stored in TE buffer and was diluted 20-fold with phosphate buffer before irradiation to reduce the ^•^OH scavenging capacity to a level estimated to be 417-fold smaller than in cells [167]. A 2.6-fold reduction in the yields of SSB induction compared with conventional dose rate (0.167 Gy/s) was found after FLASH irradiation with 16 MeV electrons (5 µs pulses, 0.518 Gy/pulse, 90 or 180 pps) giving 20 Gy at 46.6 or 93.3 Gy/s over a total time of 0.43 s and 0.216 s, respectively. The induction of DSBs also showed a trend for a reduction in yields but DSBs were observed in only 1–2% of the plasmids and yields were too low to draw firm conclusions. The sparing effect of FLASH under aerated conditions and in the absence of glutathione or similar antioxidants was suggested to be due to recombination of ^•^OH radicals with increased diffusion distances in the absence of ^•^OH radical scavengers. However, even with an estimated 417-fold longer lifetime of the ^•^OH radicals in the buffer compared with the cellular environment, the lifetime would still be of the order of 1 µs (i.e., shorter than the pulse length of 5 µs and the total time for dose delivery). Thus, ^•^OH radicals will decay within the pulse via reactions with the buffer or recombination with e_aq_^−^, and will certainly have decayed when the next pulse is given 5.5 or 11 ms later. As discussed in a previous section, recombination of ^•^OH radicals produced in different pulses cannot occur inside cells at dose rates currently achieved with FLASH irradiation, questioning the proposed mechanism of ^•^OH recombination causing the reduced SSB yields after irradiation with FLASH dose rates. An alternative explanation based on long-lived DNA radicals is presented in the section “Potential flash mechanisms in vitro and in vivo” (below).

### Protective effects of UHDR on DNA damage induced by irradiation of cells in vitro

Induction of DNA strand breaks inside cells may be studied by single-cell gel electrophoresis (comet assay) or DNA repair foci. The alkaline comet assay allows the application of typical FLASH radiation doses and detects different types of breaks but because SSBs are induced at 25-40-fold higher rates than DSBs, the vast majority of the lesions will be SSBs and alkaline-labile sites. With this assay, a significant oxygen-dependent decrease of DNA damage in peripheral blood lymphocytes was found after FLASH irradiation with 20 Gy of 6 MeV electrons when the oxygen concentration was in the range of 0.25–0.5% but not when in the range of 3–21%. Interestingly, 1% O_2_ gave intermediate results [168]. This strongly suggests that the oxygen fixation hypothesis with competition between oxygen fixation and chemical restitution of DNA radical sites by H-atom transfer from GSH and other antioxidants may underlie this type of FLASH protection. Maximum reduction of the detected lesions occurred at MDR 30 Gy/s and higher with a half maximum effect at approximately 10 Gy/s, implying a maximum overall irradiation time of 0.67 s to achieve the full sparing effect of UHDR after a dose of 20 Gy in this system.

The total number of γH2AX foci was unchanged after FLASH versus CONV irradiation of human fibroblasts and A549 lung adenocarcinoma with a single dose of 5.2 Gy 4.5 MeV electrons. However, the number of 53BP1 foci was lower after FLASH irradiation in the fibroblast lines but not in A549 cells [169], suggesting that a subset of DSBs was decreased by UHDR irradiation in fibroblasts. If UHDR reduces the complexity of DSBs (e.g., by restituting associated SSBs or base damage), this would not change the number of DSBs but would influence repairability and the choice of repair pathway. This might explain the different effects on 53BP1 foci in normal fibroblasts and the tumour cell line.

A recent study irradiated spheroids at 4 °C versus 37 °C to eliminate metabolic oxygen consumption and found a significant protective effect of FLASH (>50 Gy/s) for NHEJ-related pDNA-PK foci at 40–50 Gy when irradiated at 4 °C under 1% O_2_ and incubated for 2.5 h after irradiation [170]. However, no difference was observed for γH2AX foci except perhaps a trend under 0.2% O_2_. In metabolically active cells at 37 °C, a protective FLASH effect was only found in outer spheroid cells irradiated with 50 Gy under 3% O_2_. The authors concluded that well oxygenated cells may benefit more from FLASH-induced oxygen depletion than already hypoxic cells but the doses were high and the mechanistic interpretation was confounded by the combined effects of induction and repair of DSBs.

## Potential flash mechanisms in vitro and in vivo

Although the very early studies on UHDR irradiation may have been influenced by experimental limitations, numerous later studies have found various effects of UHDR in vitro and in vivo although sometimes only under low oxygen conditions. However, effects seen in vitro and in vivo are not necessarily caused by the same mechanisms and a better understanding of FLASH mechanisms will be necessary for optimal application in clinical radiotherapy. In particular, potential mechanisms should be able to explain not only the effect of UHDR versus CONV but also the differential protective effect of FLASH in normal tissue versus tumours. From a mechanistic point of view, the time ranges of reactions and processes are important and will be considered in the following.

### DNA radicals: restitution of molecular structure or damage fixation by oxygen

The induction, recombination and reactions of radiation-induced aqueous radicals at early timepoints <0.1 µs have been described in previous sections. In cells, recombination of ^•^OH radicals from different tracks is not possible at doses used in FLASH whereas ^•^OH may conceivably recombine with longer-lived e_aq_^−^ surviving track expansion to react on the timescale ≥1 µs. By contrast, DNA radicals formed by indirect or direct action have much longer lifetime with a first halflife ∼1 ms in the cellular environment and complete decay within 10 ms after which it can no longer react with oxygen [58]. Thus, damage fixation by reaction with O_2_ must take place at shorter times (10 ns-10 ms) to achieve radiosensitisation in competition with chemical restitution by H-atom transfer from thiols which dominates under hypoxia. In vivo, ascorbate (vitamin C) may contribute to restitution while NO may contribute to damage fixation.

**Fig. 2:**
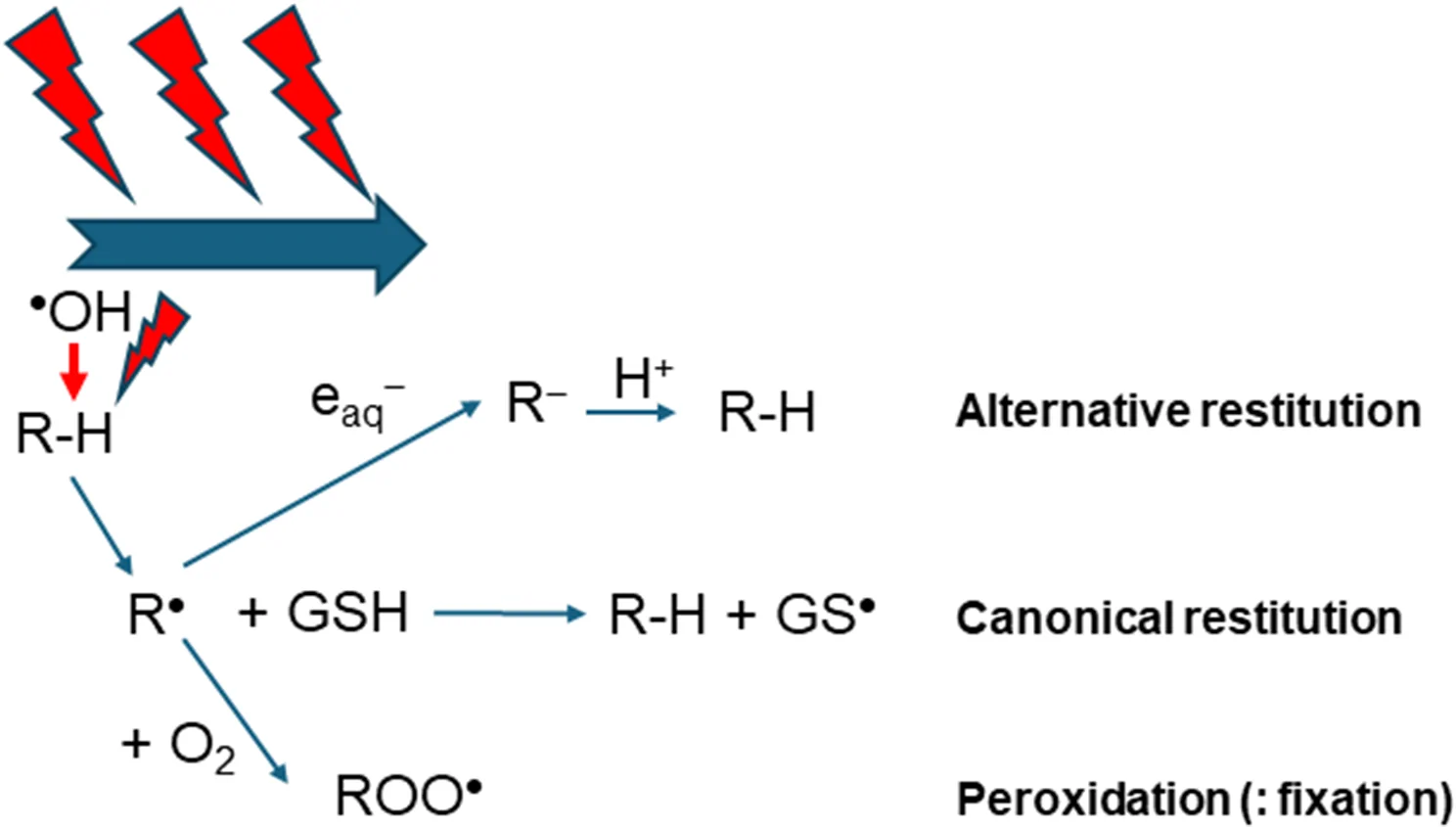
Reactions of radiation-induced DNA radicals, R^λ^, with a first half-life of ∼1 ms in cells. Canonical restitution by H-transfer from thiols (e.g., GSH) competes with O_2_ forming a peroxyradical. Alternative restitution by single-electron transfer followed by proton transfer (SET-PT) should be faster as e_aq_^−^ is a highly reducing agent and very reactive towards radicals. Fixation by reaction with O_2_ must occur within a few ms

As described earlier, e_aq_^−^ is a highly reducing species which reacts rapidly with radicals but has a low reaction rate towards DNA and a much longer lifetime than the ^•^OH radical. If the lifetime of e_aq_^−^ is of the order of 1 µs inside the nucleus, it would live long enough to escape the tracks while competing reactions prevent its reaction with O_2_ to form O_2_^−^. We suggest that, under UHDR, e_aq_^−^ escaping the spurs after track expansion may react under homogeneous kinetics with long-lived DNA radicals, produced at some distance by earlier tracks, in a single-electron transfer followed by proton transfer (SET-PT reaction). Thus, a DNA anion is formed (R^•^ + e_aq_^−^ → R^−^) which can be neutralised by H^+^ thereby restituting the molecular deoxyribose structure of DNA in competition with damage fixation by O_2_, similar to restitution by H-atom transfer. Dose rate-dependent protection by radiation-induced e_aq_^−^ may be termed ‘alternative restitution’ as opposed to the ‘canonical restitution’ by H-atom transfer from thiols (Fig. 2).

While it cannot be completely excluded that longer-living e_aq_^−^ react with ^•^OH by inter-track recombination (^•^OH + e_aq_^−^ → OH^−^), the lifetime of oxygen-sensitive radical lesions in DNA (R^•^) is six orders of magnitude longer than the lifetime of ^•^OH (ms versus ns, respectively) implying an increased concentration that should favour the reaction of e_aq_^−^ with DNA radicals (R^•^ + e_aq_^−^ + H^+^ → RH). Furthermore, the competition for e_aq_^−^ from O_2_ is decreased by physoxia/hypoxia.

The contribution from alternative restitution will depend on the e_aq_^−^ diffusion distance which can be determined by the Einstein-Smoluchowski equation x = √(τ⋅6⋅D) with a diffusion constant, D = 4.75–4.9 cm^2^s^− 1^ [171, 172]. For e_aq_^−^ lifetimes in the range, τ = 1–4 µs, the diffusion distance should be ∼170–340 nm, implying that the electron can escape the track and react with DNA radicals induced by previous tracks in the same pulse within that distance. As discussed previously, low-LET radiation deposits approximately 1000 electron tracks with ∼100 ionisations each, in a cell nucleus per Gy [35, 125], corresponding to a mean distance between tracks of the order of 240 nm for a DPP of 1 Gy and close to 100 nm for 5 Gy. However, because of the stochastic distribution of tracks, a proportion of neighbouring tracks will be closer to each other than the mean distance. Thus, the combination of a relatively long e_aq_^−^ lifetime and high dose per pulse is predicted to be critical for alternative restitution. As the first half-life of DNA radicals is of the order of 1 ms, their lifetime will only allow a minor fraction from one pulse to remain when the next pulse is given 2.5–10 ms later (for pulse repetition rates 100–400 pps). Therefore, this effect is predicted to depend mainly on the dose rate in the pulse and less on the MDR, especially for low pulse repetition rates. Notably, alternative restitution may occur when pulse lengths are shorter than the lifetime of e_aq_^−^ since the radicals from different tracks may react throughout their lifetime. By contrast, if the lifetime of e_aq_^−^ is much shorter than the pulse length, or if a low DPP results in longer distances between neighbouring tracks, the contribution from alternative restitution will become negligible.

Since free radicals have longer lifetimes in dilute aqueous solutions with low scavenging capacity, they will diffuse longer distances and, therefore, alternative restitution might explain the substantial protection of SSBs in DNA irradiated in solution at UHDR in the absence of thiols [167]. Furthermore, a recent in vivo study by Liu et al. examined FLASH sparing for different combinations of MDR and DPP for a given total dose (11–14 Gy) of 9 MeV electrons to the mouse abdomen [173]. At constant MDR (104–184 Gy/s), FLASH sparing effect on mouse crypts increased with DPP in the range 1–6 Gy per pulse whereas, for two pulses of high DPP (4.7–6.0 Gy/pulse), it was independent of the time interval between pulses (0.008–34 s) corresponding to MDR = 0.3–1440 Gy/s. For survival, three 4.7 Gy pulses given over an overall time of 0.13–45 s provided similar sparing (i.e., independent of MDR: 104 Gy/s or 0.3 Gy/s), whereas three sets of 5 pulses of 0.93 Gy per pulse given in the same temporal pattern only increased survival for the high (FLASH) MDR (104 Gy/s) but not the low (‘conventional’) MDR (0.3 Gy/s). Although no mechanism was proposed, doses of 4.7–6 Gy per pulse should be large enough to generate high local concentrations of e_aq_^−^, and reduce the distance between tracks, consistent with enhanced alternative restitution within each pulse, whereas 0.93 Gy per pulse may be too low. On the other hand, a previous study by Ruan et al., found increased sparing at high DPP (6.25 Gy/pulse) when two pulses were separated by a 0–10 ms interval but not when the time interval was 0.04–30 s [174]. While this seems consistent with the time range for competition between canonical restitution and fixation of DNA radicals by oxygen [58], the difference in sparing between the two studies for longer time intervals between pulses, and the potential role of alternative restitution, will require further studies.

Chemical restitution of DNA, whether canonical or alternative, would not need to completely prevent a DSB by simultaneously restituting breaks on opposite strands but might act by reducing the complexity of DSBs, thereby increasing their repairability and influencing the repair pathway choice. Low pH would be predicted to increase the molecular restitution of the deoxyribose anion by H^+^ but although tumours are generally more acidic than normal tissue, this affects only the extracellular microenvironment whereas the intracellular pH in cancer cells is even slightly more alkaline than in normal cells [175, 176]. The pH in the cell nucleus is less well known [175] but histone acetylation regulates pH which is modulated through the different cell-cycle phases [177, 178]. Notably, a radiation-induced transient pH decrease in water has been described [179] so it seems possible that UHDR irradiation may cause temporary intracellular acidification before this can be neutralised by cellular buffer systems.

In the presence of oxygen, ^•^O_2_^−^ is formed with lifetimes in the ms range but, as argued by Michael et al., ^•^O_2_^−^ is unlikely to sensitise cells by reaction with radiation-induced radicals on DNA [92]. On the other hand, although ^•^O_2_^−^ is predominantly an oxidising species, it also has reducing properties towards strongly oxidizing species so molecular restitution of DNA radicals by donation of the electron seems conceivable. However, this would depend on the electron affinity of the DNA radical and the concentration of radiation-induced ^•^O_2_^−^ which may be lower in cells than in dilute aqueous solutions, as argued by Wardman [12].

Although our review focuses on FLASH effects with low-LET radiation, any hypothetical mechanism should of course also be viewed in the context of FLASH irradiation with other radiation qualities. FLASH effects have been studied for heavy particles, primarily energetic protons and recently also carbon and other ions [7, 180–183]. However, the interpretation of results from high-LET FLASH studies is complicated by a wider range of dose delivery variables depending on the pulse structure related to the production method (cyclotrons or synchro-cyclotrons in the case of protons, synchrotrons in the case of heavier ions), the LET and track structure, (depending on particle mass, charge and energy) as well as beam modulation (passive or pencil beam scanning), as reviewed by Diffenderfer et al. [182]. For high-LET radiations, one might expect direct radiation action by the core of a high-LET track to dominate the induction of damage to biomolecules. However, the low-energy electrons that make up the penumbra radiating out from the track core damage biomolecules in much the same way as electron track ends from low-LET radiation with a strong contribution from indirect action. As shown in previous sections, the ^•^OH radicals from early expanding tracks do not overlap at clinically relevant doses, whereas longer-living radicals such as e_aq_^−^ may undergo the same type of reactions as described above for low-LET irradiation. The extent to which direct action dominates the induction of damage to DNA and other biomolecules will depend on the track structure but indirect action may be expected to contribute substantially to damage by proton therapy beams with moderate LET values < ∼5 keV/µm and relative biologic effectiveness (RBE) of approximately 1.1, i.e. similar to that of orthovolt radiation using ^60^Co γ-rays or MeV electrons with typical LET values of 0.2 keV/µm as reference radiation.

In contrast with proton beams, direct radiation action is likely to dominate for carbon ion beams which also show a much smaller oxygen effect. Recently, a protective effect of UHDR on clonogenic cell survival was found after 6.5 Gy of carbon ions with LET = 19 keV/µm and 50 keV/µm under 4% and 21% O_2_, which was associated with reduced numbers of induced γH2AX foci [184]. However, the correlation between residual foci after repair and clonogenic cell inactivation was not determined. Rat prostate tumour xenografts irradiated with carbon ions in the spread-out Bragg peak (dose-averaged LET = 75 keV/µm) showed a 20% higher RBE for irradiation under oxic conditions (breathing 97.5% O_2_) compared with hypoxic conditions (clamped tumours) [185, 186]. Although this is consistent with a decreased OER for carbon ions compared with 6 MV X-rays, only approximately half of the oxygen effect was eliminated by C-ions, showing that oxygen needs to be taken into account even for tumours in the spread-out Bragg peak of carbon beams. Thus, further studies are needed to characterise the role of oxygen for the FLASH effect in the entrance beam and the spread-out Bragg peak of protons and heavy ions.

### Dynamics of oxygen consumption and reoxygenation in vitro and in vivo

The bulk of experimental evidence obtained with cells and in tissues show more stable, protective UHDR effects at low oxygen concentrations in vitro and in vivo. The absolute amount of radiolytic oxygen consumption is not very different but occurs much faster for UHDR than CONV [142] which seems to suggest that dynamic differences regarding oxygen consumption and/or reoxygenation may be involved.

Most studies on UHDR irradiation with electrons in the FLASH era have used modified linear accelerators giving electron pulses of 1–3 µs length and doses of 1–3 Gy per pulse at repetition rates of 100–400 pps. Typically, this delivers doses of 10–30 Gy within 10–100 ms with 2.5–10 ms between individual pulses [169, 187] although up to 5 Gy/pulse with 3.3 ms between pulses has been reported, allowing 20 Gy to be delivered in 10 ms [168], . In such studies, oxygen levels in cells will be determined by the balance between the oxygen consumption (by metabolism and irradiation, combined) and reoxygenation from oxygen in the surroundings.

Early measurements of oxygen consumption by live cells yielded 27⋅10^− 18^ mol/s per cell [188] but later studies have revealed a wide range from 1-120⋅10^− 18^ mol/s per cell in different cell lines and even higher consumption in hepatocytes [189]. For a sparse cell culture (e.g., 2.5⋅10^5^ cells and 5 ml medium in a T25 cell culture flask), an average value of 60⋅10^− 18^ mol/s per cell translates into an oxygen concentration consumption rate of 0.003 µM/s. Although metabolic consumption may influence steady state oxygen levels, the associated change in oxygen concentration during FLASH irradiation lasting less than a second should be negligible. The dynamics of oxygen gradients after transfer to hypoxic conditions has been studied in stirred cell suspensions [140] and recently in relative large volumes of medium in tissue culture flasks [190]. The latter study showed that while it may take one to several hours to reach equilibrium under hypoxia, reoxygenation occurs within seconds or minutes. Diffusion through barriers with a concentration gradient is governed by Fick’s law and, for 2 mm medium above cells in standard culture conditions, is likely to occur within a few seconds. For radiation-induced oxygen consumption in less than about a second, diffusion of oxygen from the gas phase will be too slow for reoxygenation during irradiation and a transient, partial oxygen depletion will occur.

**Fig. 3:**
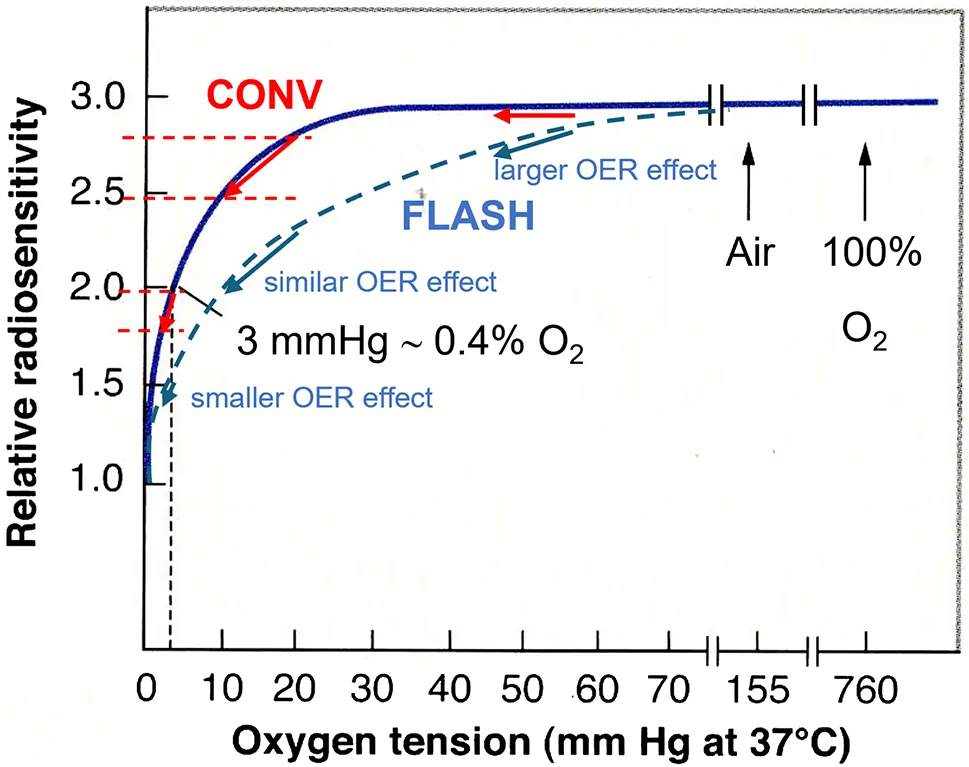
Schematic representation of the relative radiosensitivity (oxygen enhancement ratio, OER) as a function of oxygen tension showing a steep increase under hypoxia with half maximal effect at 3 mmHg. (modified from Hall, Giaccia, 2019 [35]). The solid curve is based on experimental data and the oxygen fixation hypothesis in competition with H-atom transfer from intracellular thiols (mainly GSH) for irradiation at conventional dose rate. The red arrows signify approximately 50% transient reduction in oxygen levels by FLASH irradiation with 20 Gy at 3–20 mmHg and a reduction by 10 mmHg at higher initial oxygen tensions. Because of the high reducing reactivity of e_aq_^−^, alternative restitution by single-electron transfer followed by H-transfer (SET-PT) should be faster than H-transfer from GSH, leading to a displacement of the curve towards higher oxygen concentrations (broken curve). As a result, oxygen consumption by FLASH irradiation should have a larger effect on OER at higher initial tension but a lower effect under hypoxia (blue arrows)

Under physiological conditions in the body, most normal tissues maintain an oxygen concentration in the range 3–7% (23–55 mmHg) whereas the oxygen concentration in tumours is in the range 0.3–4.2% (2–32 mmHg) [191]. The radiobiological oxygen effect on cell inactivation increases the radiosensitivity with increasing oxygen tensions in the range of 0–30 mmHg (approx. 4% or 40–50 µM O_2_). At 3 mmHg, the oxygen enhancement ratio (OER) is half its maximum value which is reached asymptotically at concentrations above 40–50 mmHg (Fig. 3) [35].

If it is accepted that radiation-induced oxygen consumption per Gy is nearly proportional to the initial oxygen concentrations up to 10–20 mmHg as suggested by recent studies [120, 145–148, 150], 40–50% depletion may be expected after a typical FLASH dose of 20 Gy. At higher initial concentrations, the consumption per Gy is nearly constant resulting in a decrease of 9–10 mmHg after 20 Gy. Partial oxygen depletion may still protect cells but the protective effect may be smaller at decreasing initial concentrations although the steeper slope of the OER curve may compensate for this (Fig. 3). However, e_aq_^−^ is a much stronger reducing agent against radicals than H-atom transfer from thiols, so the OER curve versus oxygen tension for alternative restitution will be displaced to the right relative to the curve for canonical restitution. As a result, the reduction in OER caused by a given radiation-induced oxygen consumption will be larger at higher initial oxygen tensions and smaller at lower initial oxygen tensions compared with canonical restitution. Thus, the alternative restitution combined with lower initial oxygen concentrations in tumours than in normal tissue may contribute to the differential FLASH effect in normal tissue relative to tumours. Furthermore, the increased scavenging capacity from nucleosides/nucleotides in cell nuclei during replication (cell cycle S-phase) should decrease the lifetime of e_aq_^−^ thus providing less alternative restitution in rapidly proliferating tumour cells compared with more slowly proliferating or quiescent normal cells.

It seems uncertain if alternative restitution can explain the partial protection at intermediate dose rates in the pulse (3⋅10^3^ − 6⋅10^4^ Gy/s) observed by Hendry et al. [134]. In this range, rat tails were irradiated with relatively low doses per pulse (0.002–0.055 Gy) implying that the number of tracks per nucleus was lower (2–55), and the distance between tracks correspondingly larger. Furthermore, increased protection with increasing pulse dose rate was not observed in the range 3⋅10^3^ − 6⋅10^4^ Gy/s. On the other hand, the two mouse studies on regenerating intestinal crypts showed clear sparing effects of large DPP. In the study by Liu et al., this effect was observed even when the interval between pulses was 22.5 s [173]. Since some reoxygenation may be expected over this time interval, these results may support the hypothesis of alternative restitution. In the study by Ruan et al., the strongest sparing effect was observed for a single pulse of 12.5 Gy, less sparing for two pulses of 6.25 Gy separated by 3–10 ms, and none for longer intervals [174]. This seems more consistent with partial oxygen depletion and canonical restitution competing with fixation by oxygen. Another caveat is that if alternative restitution were associated with slowly dividing cells, a sparing effect on intestinal stem cells might not be expected. Furthermore, tumour growth delay after three pulses of 6.7 Gy per pulse showed no FLASH sparing irrespective of whether the interval between pulses was 8–30 s but the total dose (20 Gy) was considerably higher than for the intestinal endpoints [173] and physiological oxygen levels may be different. Clearly, more studies are needed on the roles of DPP and alternative versus canonical restitution.

In well-perfused normal tissues in vivo, cells are typically closer than 100 μm to a capillary vessel. Modelling oxygen diffusion in tissue supplied by a capillary network is complicated (reviewed by Goldman [192]) but, as discussed previously, diffusion times for a distance of 50–100 μm should be in the range 0.4–5 s which will be too slow for reoxygenation during FLASH irradiation with a duration of typically less than 0.5 s whereas continuous reoxygenation will be possible during irradiation at conventional dose rate. Most normal tissues are mildly hypoxic in normal physiological conditions, and although local variations are predicted [192], the oxygen concentration is probably more uniform than in tumour tissue. Changes in perfusion by NO-mediated vasodilation may contribute to reoxygenation but this is a slower process occurring over many seconds or minutes.

Many tumours are characterised by immature, poorly regulated vasculature that may be leaky with a heterogenous mix of well oxygenated areas and areas with acutely or chronically hypoxic cells (completely anoxic cells die from necrosis) [193]. Assuming a binary mixture of radiobiologically hypoxic and fully oxygenated cells (< 0.5 mmHg and > 40 mmHg O_2_, respectively), the tumour response at higher doses is predicted to be determined by the fraction of resistant, hypoxic cells after the more sensitive cells have been killed at lower doses [35]. In many tumours, however, the majority of cells will be at an intermediate partial pressure of 0.5–20 mmHg, (i.e. the range where the radiobiological oxygen effect changes from protection to sensitisation). A biologically more realistic model where diffusion from capillaries at physiological oxygen concentration into the tissue was taken into account, suggested that the cell survival curve is much closer to that of resistant hypoxic cells than previously expected and that the tumour reponse may be determined largely by this cell population [194]. In this case, partial oxygen depletion by FLASH would not be expected to cause further radioprotection and reoxygenation during irradiation at conventional dose rate would not be relevant either. Taken together, 40–50% oxygen depletion by FLASH irradiation should be able to reduce the oxygen concentration of well-oxygenated normal cells under physoxia and move them into the intermediate range to cause radiobiological protection whereas partial oxygen depletion of tumour cells already at intermediate oxygen concentrations may not cause significant further protection. Thus, from a physiological and radiobiological point of view, transient oxygen ‘depletion’ may be responsible for the differential protective effect of FLASH in normal tissue compared with tumours, even though depletion is only partial and not complete [195].

The rat tail necrosis model may differ from other normal-tissue endpoints by having a reduced blood flow at the lower ambient temperature. Nevertheless, studies on protective effects of UHDR/FLASH irradiation in mouse intestine and brain clearly also indicated reduced physiological oxygen levels [119, 136], lending support to the notion of limited perfusion of blood capillaries. Radiobiological cell death is likely to play a major role for tail necrosis and lethal intestinal breakdown which are early reactions (scored at week 7 and day 5, respectively). This differs from late normal-tissue reactions such as fibrosis which is thought to develop by complex mechanisms involving interactions between different cell types, including immune cells, via pro- and anti-inflammatory cytokines and pro-fibrogenic growth factors [196–198]. This contrasts with the radiation-induced inactivation of tumours which is related to the radiosensitivity of tumour cells although immunogenic cell death also contributes [199, 200].

The oxygen consumption for FLASH irradiation with proton and heavy ion beams was studied as function of LET by Karle et al. [150] over the range 1-100 keV/µm (for 9 MeV electrons used as low-LET radiation, a value of 1 keV/µm was given although 0.2 keV/µm should be more appropriate). An approximately two-fold decrease with increasing LET was ascribed to increasing intra-track radical recombination with increasing production of oxygen in the track playing only a minor role. Furthermore, the study confirmed a slightly lower consumption at UHDR compared with CONV dose rate as observed for low-LET radiation. However, the slow response for equilibration of the OxyLite™ sensor with the surrounding oxygen concentration prevented a time resolution of the dynamic oxygen consumption between UHDR (108–121 Gy/s) and CONV (0.31–0.40 Gy/s), with dose delivery times of 0.125–0.138 s and 36.8–48.5 s, respectively. As discussed in the previous section, more studies are needed on the role of oxygen and its dependence on LET in the entrance beam which is more relevant for normal-tissue reactions and the spread-out Bragg peak which is more relevant for tumours in the target volume.

### Potential role of mitochondria

The main organelles for redox processes in cells are the mitochondria which perform oxidative phosphorylation and act as the major oxygen sink in cellular physiology. To date, few studies have investigated the role of mitochondria in the FLASH effect. However, a study by Guo et al. [201] showed that the mitochondrial damage and dysfunction in human fibroblasts induced by irradiation of normal human fibroblasts (IMR90) with protons at (relatively high) CONV dose rate (0.33 Gy/s) under ambient oxygen tension was prevented when irradiation was performed at FLASH dose rate (100 Gy/s). Thus, irradiation at FLASH dose rate abrogated upregulation of ROS levels, a decrease in mitochondrial length, and strong upregulation of the GTPase, Drp1 (dynamin-related protein 1), which regulates mitochondrial fission, and co-localised with tumour suppressor protein p53. This was accompanied by strong downregulation of proteins involved in cell growth and death decisions (PTEN, cytochrome c, c-myc, autophagy proteins LC3A and LC3B) at CONV but not FLASH dose rate. Furthermore, cell death by necrosis dominated over apoptosis 4 h after irradiation with conventional dose rate whereas the opposite was true for FLASH dose rate. Finally, no difference in cell viability after irradiation with CONV or FLASH dose rates was observed when Drp1 was knocked down, supporting a causal relationship of the FLASH effect with Drp1.

Oxidative phosphorylation produces energy from pyruvate derived by glycolysis in the form of ATP produced by five complexes of the electron transport chain spanning the inner mitochondrial membrane which is coupled to the tri-carboxylic acid (TCA) cycle (also known as the ‘citrate’ or ‘Krebs’ cycle) [202]. Superoxide radicals are produced when electrons leak out of the electron transport chain, especially from complex I during reverse electron transport, and mainly into the matrix [203]. The superoxide radical anion cannot diffuse through membranes but SOD2 will convert it to H_2_O_2_ which can cross the membrane. It has been hypothesised that high levels of ^•^O_2_^−^ may activate uncoupling proteins and that mild uncoupling of the electron transport chain might reduce the production of ^•^O_2_^−^ with reduced production of ATP and of increased heat production [202, 203].

In normal cells, superoxide levels are maintained at a well-regulated level. The induction of ROS by conventional and FLASH dose rates might be explained by capture of radiation-induced e_aq_^−^ by O_2_ or perhaps by radiation-induced e_aq_^−^ interfering with the electron transport chain to cause reverse electron transport. At conventional dose rate, reoxygenation would ensure a steady concentration of oxygen whereas at FLASH dose rate, a combination of recombination of e_aq_^−^ and transient oxygen consumption might prevent the radiation-induced increase in ^•^O_2_^−^. Cancer cells rely more on glycolysis without oxidative phosphorylation, leading to moderately elevated basal levels of ^•^O_2_^−^ and reduced oxygen consumption through complex IV of the electron transport chain. In the study by Guo et al., mitochondrial morphology and subcellular distribution in A549 tumour cells differed strongly from that of fibroblasts but no difference was observed between cells irradiated at conventional and FLASH dose rates, and radiation-induced changes in ROS levels were not determined [201].

Normal-tissue reactions are often considered to be associated mainly with premature differentiation or senescence of functional cells, and with interactions between different cell types, including immune cells mediating inflammatory and pro-fibrogenic reactions [196–198], whereas tumour control is more dependent on clonogenic inactivation of cancer cells. Mitochondria are thought to be involved in senescence, inflammation, and normal-tissue disease [204–206], but are not considered primary targets for clonogenic inactivation of cancer cells although they mediate apoptotic cell death by the intrinsic pathway after radiation-induced damage to nuclear DNA. Thus, experimental evidence and theoretical considerations support a role of mitochondria in the FLASH effect (e.g., via interference with ^•^O_2_^−^ production and the electron transport chain). Open questions regarding the effect of radiation-induced electrons at high local concentrations during FLASH versus lower concentrations during longer times at conventional dose rate, as well as effects in normal and cancer cells at different oxygen concentrations should be addressed in future studies.

## Conclusions

The protection by UHDR/FLASH irradiation relative to CONV dose rates may involve processes covering a wide time range from ns to several seconds or even minutes. Radiation-induced free radicals and secondary radicals, including radicals formed in DNA, have lifetimes ranging from ns to ms. Fast reactions (< 0.1 µs) occur in the expanding ionisation tracks under non-homogeneous kinetics and other reactions occurring after track expansion (≥1 µs) under homogeneous kinetics. In addition, radiation-induced consumption of oxygen occurs during irradiation, for instance, up to approximately 0.5 s for UHDR/FLASH irradiation and over one to several minutes for irradiation at conventional dose rates, while reoxygenation may take seconds or minutes and, in some cases, may involve changes in perfusion. The evidence suggests that more than one mechanism is involved with some mechanisms operating within a single (ns-µs) pulse and others over several, repeated pulses. Thus, FLASH effects may depend on the dose rate in the pulse as well as on MDR, with the dose in the pulse and the total irradiation time being important influencing factors. In many experimental settings, reduced or physiological levels of oxygen are required for protection. The oxygen fixation hypothesis explains the radiobiological oxygen effect by a competition between damage fixation of radiation-induced radicals in DNA by oxygen and molecular restitution by H-atom transfer from antioxidants such as thiols. We suggest a pathway for alternative restitution mediated by radiation-induced aqueous electrons that may contribute to protection within individual pulses of irradiation at UHDR.

## Data Availability

No datasets were generated or analysed during the current study.

## Footnotes

1 Chemical reactions take place with non-homogeneous (heterogenous) kinetics when the constituents are not uniformly distributed or are in two different phases (e.g. solution and the surface of a solid, respectively). The reaction of non-homogeneously distributed ^•^OH radicals with DNA in solution may be modelled as the reaction of diffusing ^•^OH radicals with the surface of a solid cylinder (Udovicic L, Mark F, Bothe E, Schulte-Frohlinde D., Int J Radiat Biol. 1991;59 (3):677−97).
